# Comparative mapping of *Brassica juncea *and *Arabidopsis thaliana *using Intron Polymorphism (IP) markers: homoeologous relationships, diversification and evolution of the A, B and C Brassica genomes

**DOI:** 10.1186/1471-2164-9-113

**Published:** 2008-03-03

**Authors:** Priya Panjabi, Arun Jagannath, Naveen C Bisht, K Lakshmi Padmaja, Sarita Sharma, Vibha Gupta, Akshay K Pradhan, Deepak Pental

**Affiliations:** 1 Centre for Genetic Manipulation of Crop Plants, University of Delhi South Campus, Benito Juarez Road, New Delhi 110021, India; 2 Department of Genetics, University of Delhi South Campus, Benito Juarez Road, New Delhi 110021, India; 3 National Institute for Plant Genome Research, Aruna Asaf Ali Marg, New Delhi 110067, India

## Abstract

**Background:**

Extensive mapping efforts are currently underway for the establishment of comparative genomics between the model plant, *Arabidopsis thaliana *and various Brassica species. Most of these studies have deployed RFLP markers, the use of which is a laborious and time-consuming process. We therefore tested the efficacy of PCR-based Intron Polymorphism (IP) markers to analyze genome-wide synteny between the oilseed crop, *Brassica juncea *(AABB genome) and *A. thaliana *and analyzed the arrangement of 24 (previously described) genomic block segments in the A, B and C Brassica genomes to study the evolutionary events contributing to karyotype variations in the three diploid Brassica genomes.

**Results:**

IP markers were highly efficient and generated easily discernable polymorphisms on agarose gels. Comparative analysis of the segmental organization of the A and B genomes of *B. juncea *(present study) with the A and B genomes of *B. napus *and *B. nigra *respectively (described earlier), revealed a high degree of colinearity suggesting minimal macro-level changes after polyploidization. The ancestral block arrangements that remained unaltered during evolution and the karyotype rearrangements that originated in the Oleracea lineage after its divergence from Rapa lineage were identified. Genomic rearrangements leading to the gain or loss of one chromosome each between the A-B and A-C lineages were deciphered. Complete homoeology in terms of block organization was found between three linkage groups (LG) each for the A-B and A-C genomes. Based on the homoeology shared between the A, B and C genomes, a new nomenclature for the B genome LGs was assigned to establish uniformity in the international Brassica LG nomenclature code.

**Conclusion:**

IP markers were highly effective in generating comparative relationships between *Arabidopsis *and various Brassica species. Comparative genomics between the three Brassica lineages established the major rearrangements, translocations and fusions pivotal to karyotype diversification between the A, B and C genomes of *Brassica *species. The inter-relationships established between the Brassica lineages vis-à-vis *Arabidopsis *would facilitate the identification and isolation of candidate genes contributing to traits of agronomic value in crop Brassicas and the development of unified tools for Brassica genomics.

## Background

Extensive genome sequencing and genetic mapping studies have been performed on members of the Brassicaceae family which contains the most widely studied model species, *Arabidopsis thaliana *(At) and many economically important vegetable and oilseed crops belonging to the genus *Brassica*. An avowed goal of structural and functional genomics of At is to develop improved strategies for precision breeding of crop plants related to the model species. Since the genome size of Brassica species (529–696 Mb for the diploids and 1068–1284 Mb for the polyploids) [1] is much larger than that of At (125 Mb), there is a high probability that novel gene interactions have evolved in the Brassicas through the processes of sub-functionalization and/or neo-functionalization of paralogs [2-4]. Comparative mapping between At and Brassica species, coupled with the base knowledge of mutation-based functional analysis in At and QTL mapping in crop Brassicas, could greatly contribute towards a better understanding of the genetic architecture for the conserved as well as the evolved traits of agronomic value in the Brassicaceae.

The three diploid *Brassica *species, *B. rapa *(n = 10, AA), *B. nigra *(n = 8, BB) and *B. oleracea *(n = 9, CC) and the two allopolyploids, *B. napus *(AACC) and *B. juncea *(AABB), have been subjected to extensive genetic mapping using molecular markers to identify loci associated with various qualitative and quantitative traits of agronomic interest [5-12]. Some of the mapped quantitative traits like pod size, pod number, pod density, seed size, seed number per pod and oil content are of great importance in improving the yield of the oilseed Brassica species [11,13,14].

Recent attempts to develop a unified comparative genomics system in the Brassicaceae has recognized the existence of 24 conserved genomic blocks [15] which is an extension of 21 syntenic blocks identified in *B. napus *in an earlier study [16]. Comparative mapping studies between members of family Brassicaceae [16-20], At and *Arabidopsis lyrata *[21], At and *Capsella rubella *[22] and the identification of an ancestral karyotype (AK) [23] have also stimulated interest in the evolutionary processes involved in the diversification of different lineages in the Brassicaceae and variations in chromosome number of different species vis-à-vis their ploidy status. Most of the earlier studies on comparative mapping in Brassica species [16,18-20,24] have relied on the use of RFLP markers. However, the deployment of RFLP markers in large segregating populations is a rather cumbersome and laborious process. In recent years, several studies have highlighted the immense potential of polymorphisms in intron sequences for the development of markers for genetic mapping [25-27].

In the present study, we have successfully used PCR-based Intron Polymorphism (IP) markers for the development of a comparative map between *B. juncea*, related Brassica species (*B. napus *and *B. nigra*) and At. We also analyzed the segmental structure of the A and B genomes of *B. juncea *in terms of the 24 genomic blocks (A-X) proposed earlier [15]. We compared colinearity of the *B. juncea *and At genomes and also analyzed synteny between the A, B and C genomes of the *Brassica *species. Additionally, homoeologous linkage groups of the three genomes were identified. On the basis of homoeology among the three genomes, we propose a re-designation of the B genome linkage groups assigned earlier for *B. nigra *[18] and *B. juncea *[11]. The comparative map between *B. juncea *and At developed in this study, in conjunction with the extensive information available from functional genomics studies of the At genome, will greatly facilitate the identification of candidate genes and novel gene interactions responsible for the domestication and evolution of the yield influencing traits in *B. juncea *and other crop Brassicas.

## Results

### Comparative map of *B. juncea *and *Arabidopsis thaliana *(At) using Intron Polymorphism (IP) markers

Single copy genes from Arabidopsis (At) [28], physically located at an approximate distance of 100–200 kb, were used to design PCR primers spanning intronic sequences. In cases where large genomic regions were devoid of single copy genes, primers were designed from multiple copy genes. Primers for PCR amplification were designed from exon sequences which showed strong nucleotide conservation between At and the corresponding EST or GSS sequences described for any *Brassica *species [29].

**Figure 1:**
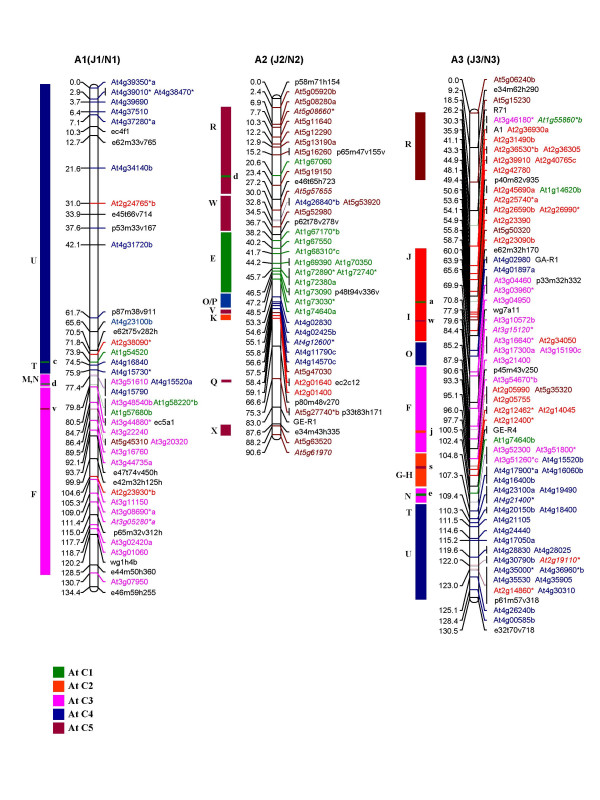
Genetic map of *B. juncea *showing three linkage groups of the A genome (A1, A2 and A3). The corresponding nomenclature followed earlier in *B. juncea *(J1–J3) [11] and *B. napus *(N1–N3) [16] is given in parentheses. Each genetic locus bears the name of the At (*A. thaliana*) gene and the colour code of the At chromosome from which it is derived. At loci in italics represent the RFLP probes mapped earlier [11]. Loci marked with an asterisk (*) are derived from multicopy At genes [28]. Loci in black represent markers of the framework map [11]. The organization of the *B. juncea *LGs based on the genomic blocks identified by Schranz et al. [15]has been represented on the left of each linkage group. The genomic blocks have been coloured differently based on the five At chromosomes from which they originate. Single copy At loci from different blocks mapped as unique insertions are shown in lower case on the right of each genomic block.

**Figure 2:**
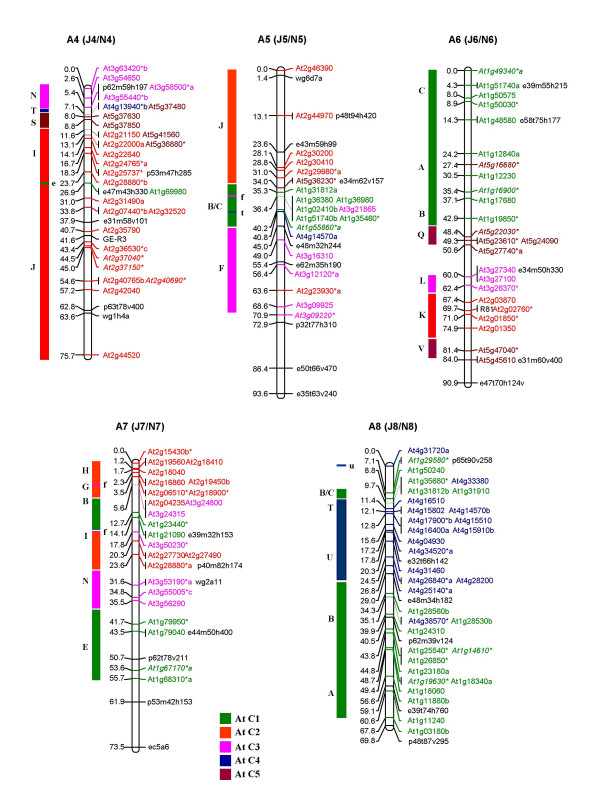
Genetic map of *B. juncea *showing five linkage groups of the A genome (A4, A5, A6, A7 and A8). The corresponding nomenclature followed earlier in *B. juncea *(J4–J8) [11] and *B. napus *(N4–N8) [16] is given in parentheses. Each genetic locus bears the name of the At (*A. thaliana*) gene and the colour code of the At chromosome from which it is derived. At loci in italics represent the RFLP probes mapped earlier [11]. Loci marked with an asterisk (*) are derived from multicopy At genes [28]. Loci in black represent markers of the framework map [11]. The organization of the *B. juncea *LGs based on the genomic blocks identified by Schranz et al. [15] has been represented on the left of each linkage group. The genomic blocks have been coloured differently based on the five At chromosomes from which they originate. Single copy At loci from different blocks mapped as unique insertions are shown in lower case on the right of each genomic block.

**Figure 3:**
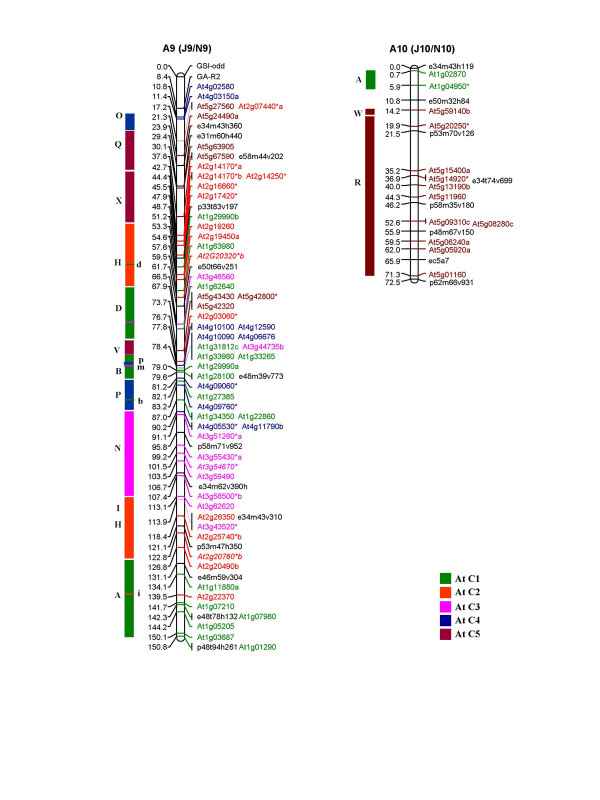
Genetic map of *B. juncea *showing two linkage groups of the A genome (A9 and A10). The corresponding nomenclature followed earlier in *B. juncea *(J9 and J10) [11] and *B. napus *(N9 and N10) [16] is given in parentheses. Each genetic locus bears the name of the At (*A. thaliana*) gene and the colour code of the At chromosome from which it is derived. At loci in italics represent the RFLP probes mapped earlier [11]. Loci marked with an asterisk (*) are derived from multicopy At genes [28]. Loci in black represent markers of the framework map [11]. The organization of the *B. juncea *LGs based on the genomic blocks identified by Schranz et al. [15] has been represented on the left of each linkage group. The genomic blocks have been coloured differently based on the five At chromosomes from which they originate. Single copy At loci from different blocks mapped as unique insertions are shown in lower case on the right of each genomic block.

**Figure 4:**
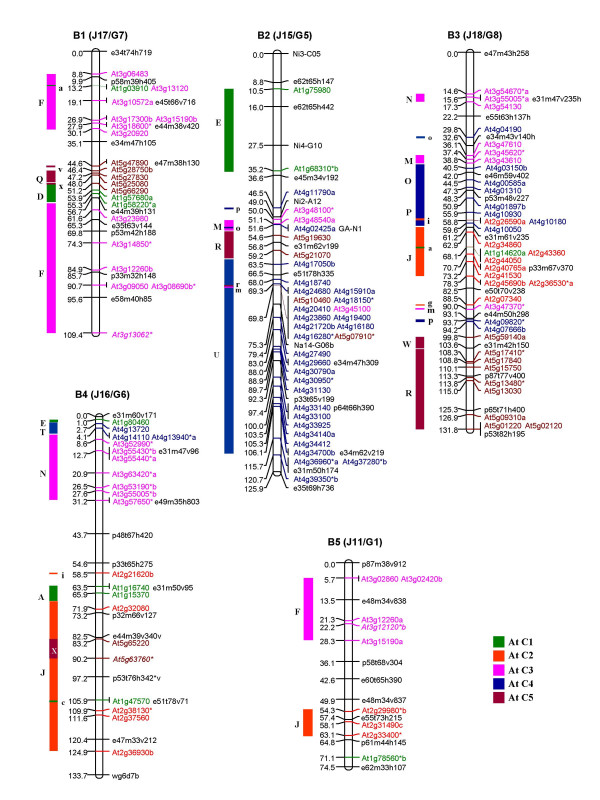
Genetic map of *B. juncea *showing five linkage groups of the B genome (B1, B2, B3, B4 and B5). The corresponding nomenclature followed earlier for the B genome of *B. juncea *(with a prefix J) [11] and *B. nigra *(with a prefix G) [18] is given in parentheses. This new nomenclature for the B genome linkage groups has been designated based on the comparative homoeology discerned between the A, B and C genomes in this study. Each genetic locus bears the name of the At (*A. thaliana*) gene and the colour code of the At chromosome from which it is derived. At loci in italics represent the RFLP probes mapped earlier [11]. Loci marked with an asterisk (*) are derived from multicopy At genes [28]. Loci in black represent markers of the framework map [11]. The organization of the *B. juncea *LGs based on the genomic blocks identified by Schranz et al. [15] has been represented on the left of each linkage group. The genomic blocks have been coloured differently based on the five At chromosomes from which they originate. Single copy At loci from different blocks mapped as unique insertions are shown in lower case on the right of each genomic block.

**Figure 5:**
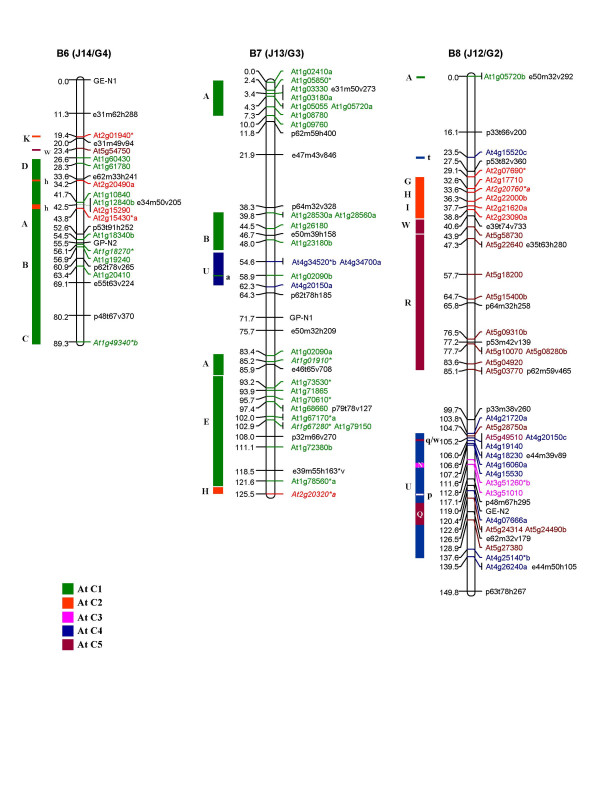
Genetic map of *B. juncea *showing three linkage groups of the B genome (B6, B7 and B8). The corresponding nomenclature followed earlier for the B genome of *B. juncea *(with a prefix J) [11] and *B. nigra *(with a prefix G) [18] is given in parentheses. This new nomenclature for the B genome linkage groups has been designated based on the comparative homoeology discerned between the A, B and C genomes in this study. Each genetic locus bears the name of the At (*A. thaliana*) gene and the colour code of the At chromosome from which it is derived. At loci in italics represent the RFLP probes mapped earlier [11]. Loci marked with an asterisk (*) are derived from multicopy At genes [28]. Loci in black represent markers of the framework map [11]. The organization of the *B. juncea *LGs based on the genomic blocks identified by Schranz et al. [15] has been represented on the left of each linkage group. The genomic blocks have been coloured differently based on the five At chromosomes from which they originate. Single copy At loci from different blocks mapped as unique insertions are shown in lower case on the right of each genomic block.

Of the 1180 primer pairs thus designed, 383 (32%) showed polymorphism between the *B. juncea *lines, Heera and Varuna, parents of the DH mapping population used in this study and in the earlier mapping studies on *B. juncea *[11,30]. Genotyping using the 383 polymorphic primer pairs (for primer sequences see Additional file 1) generated 486 loci in *B. juncea *of which 67% were scored as co-dominant markers and the remaining 33% were scored as dominant markers. These 486 loci were incorporated into the framework map developed by Pradhan et al. [30]. Additionally 34 RFLP markers placed earlier on the *B. juncea *map [11] were assigned corresponding At loci by subjecting the available sequence data to NCBI BLASTN search and identifying the most significant BLASTN hit as the source At gene. A linkage map of *B. juncea *consisting of 533 At loci (486 IP, 34 RFLP and 13 gene markers) and covering a total genetic length of 1992.2 cM is shown in Figure 1, 2, 3, 4, 5. The ten A genome linkage groups (LGs) of the *B. juncea *map were designated A1–A10 and correspond to the N1–N10 linkage groups of *B. napus *[16]. The remaining eight B genome LGs were designated B1–B8 based on homoeology between the A, B and C genomes as determined in the present study. The corresponding linkage group nomenclature proposed earlier for the *B. nigra *genome (G1–G8) [18] and the B genome of *B. juncea *(J11–J18) [11] is also given in parentheses in Figure 4, 5 and Table 1.

**Table 1:** Characteristics of *B. juncea *map based on the distribution of 533 At loci derived from the five chromosomes of *Arabidopsis thaliana*

	**At Chromosome**					**Total markers**	**Length in cM**	**Marker density (marker/cM)**	**No. of gaps (>20 cM distance)**
	**C1**	**C2**	**C3**	**C4**	**C5**				
**A genome**									
A1 (J1/N1)	3	3	13	13	1	33	134.4	0.25	1
A2 (J2/N2)	12	2	0	6	15	35	90.6	0.39	0
A3 (J3/N3)	3	21	14	24	4	66	130.5	0.51	0
A4 (J4/N4)	1	17	4	1	5	28	75.7	0.37	0
A5 (J5/N5)	7	6	5	1	1	20	93.6	0.21	1
A6 (J6/N6)	10	4	3	0	7	24	90.9	0.26	0
A7 (J7/N7)	6	12	6	0	0	24	73.5	0.33	0
A8 (J8/N8)	18	0	0	16	0	34	69.8	0.49	0
A9 (J9/N9)	17	15	9	10	7	58	150.8	0.38	0
A10 (J10/N10)	2	0	0	0	11	13	72.5	0.18	0
**Total**	79	80	54	71	51	335	982.3		2

**B genome**									
B1 (J17/G7)	3	0	13	0	5	21	109.4	0.19	0
B2 (J15/G5)	2	0	3	27	4	36	125.9	0.29	1
B3 (J18/G8)	1	9	7	10	9	36	131.8	0.27	0
B4 (J16/G6)	4	5	7	3	2	21	133.7	0.16	1
B5 (J11/G1)	1	3	5	0	0	9	74.5	0.12	1
B6 (J14/G4)	9	4	0	0	1	14	89.3	0.16	2
B7 (J13/G3)	24	1	0	3	0	28	125.5	0.22	2
B8 (J12/G2)	1	6	2	10	14	33	149.8	0.22	1
**Total**	45	28	37	53	35	198	939.9		8

**Gross Total**	**124**	**108**	**91**	**124**	**86**	**533**	**1922.2**		**10**

The figures in the parentheses are the corresponding LGs of *B. juncea *and *B. napus *[11, 16] for the A genome and the corresponding LGs of *B. juncea *and *B. nigra *[11, 18] for the B genome.

The 533 At loci mapped with variable frequencies to all the 18 linkage groups of *B. juncea *(Table 1). Linkage group A3 contained the highest number of markers (66) while B5 had the least with only 9 markers. Overall, 63% of the markers mapped to the A genome (A1–A10) at an average marker density of 0.34 and average marker interval of 2.9 cM (Table 1). The B genome (B1–B8) contained the remaining 37% of the markers with a marker density of 0.24 at an average marker interval of 4.7 cM, indicating that there is less polymorphism in the B genome as compared to the A genome in the intronic regions. As a result a number of unmapped islands were found in the B genome (an island is defined as a region with a gap of ≥ 20 cM between adjacent markers) (Table 1).

An uneven distribution of At loci originating from each Arabidopsis chromosome was observed in the genome of *B. juncea*. Among the 10 LGs of the A genome (A1–A10), all the linkage groups except A2, A6, A7, A8 and A10 contained At loci from each of the five Arabidopsis chromosomes (At C1–At C5). A2 and A6 were devoid of loci from At C3 and At C4 respectively. A7 did not contain any locus from At C4 and At C5. The linkage group A8 was composed of markers from At C1 and At C4 while A10 was composed of markers from At C1 and At C5 (Table 1). Linkage group A9 was found to be the most chimeric linkage group consisting of 12 genomic blocks followed by A3 with 10 blocks. Linkage group A10 was least chimeric with only 3 blocks followed by A5 with 4 blocks and A1 and A4 with 5 blocks each. The simplest genomic organization was observed in linkage groups A1 (primarily consisting of At C3 and At C4 loci), A4 (composed primarily of At C2 and At C3 loci), A8 (composed of At C1 and At C4 loci) and A10 (composed of At C1 and At C5 loci). Uneven distribution of the At loci was also observed in the B genome (B1–B8) of *B. juncea *of which linkage groups B3, B4 and B8 contained loci derived from all the five chromosomes of Arabidopsis (Table 1).

The organization of the *B. juncea *linkage map with respect to the At genome was also studied on the basis of the distribution of 24 genomic blocks (A-X) described for a hypothetical ancestor of the At and Brassica lineages by Schranz et al. [15]. This approach facilitated the identification of conserved blocks between At and *B. juncea*. A conserved block was defined as a region that contained at least two At loci from the same block region. In some instances, a block was recognized even with a single mapped marker if one or more markers of the same block were found mapping at the corresponding region in earlier maps for the A and B genomes [16,18]. Using this criteria, a total of 67 genomic blocks were identified in the A genome of *B. juncea *with an average of 2.8 paralogous blocks for each block recognized in the hypothetical ancestral species (Table 2). As compared to the A genome, we identified a lesser number of blocks (42) in the B genome (Table 2). Among the 42 blocks identified in the B genome of *B. juncea*, the larger blocks viz. E, F, J, R and U were observed to be represented by three paralogous blocks within the B genome (Table 2).

**Table 2:** Distribution of the 24 genomic blocks (A-X) in the A and B genomes of *B. juncea*

**Blocks**	**Gene**	**Length of conserved block (Mb)**	**A genome**	**B genome**
A	At1g01560 – At1g19330	6.4	4	5
B	At1g19850 – At1g36240	6.7	6	2
C	At1g43590 – At1g56145	4.5	3	1
D	At1g63770 – At1g56520	2.4	1	2
E	At1g65040 – At1g80420	6	2	3
F	At3g01040 – At3g25520	9.2	3	3
G	At2g05170 – At2g07733	1.6	2	1
H	At2g15670 – At2g21140	2.2	4	2
I	At2g21160 – At2g28910	3.3	4	1
J	At2g31040 – At2g47730	6.3	3	3
K	At2g01250 – At2g03750	1	2	1
L	At3g25855 – At3g29772	2.1	1	0
M	At3g43740 – At3g49970	2.8	1	2
N	At3g50950 – At3g62790	4.2	5	3
O	At4g00030 – At4g04955	2.5	3	1
P	At4g12070 – At4g08690	1.6	2	1
Q	At5g28897 – At5g22800	3.3	3	2
R	At5g22030 – At5g01240	7.1	3	3
S	At5g41900 – At5g32621	4.4	1	0
T	At4g12750 – At4g16143	1.6	4	1
U	At4g16250 – At4g38770	8.9	3	3
V	At5g48520 – At5g42970	2.4	3	0
W	At5g49430 – At5g60390	4.2	2	2
X	At5g60550 – At5g67385	2.5	2	0

**Total**		**97.2**	**67**	**42**

### Comparative block arrangement in the A genomes of *B. juncea *and *B. napus*

**Figure 6:**
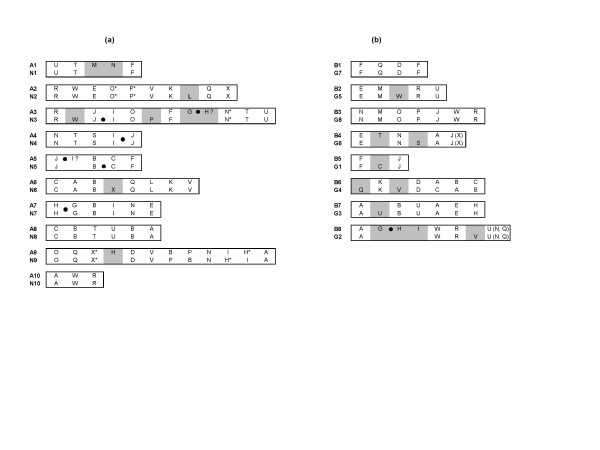
Comparative block arrangement in (a) the A genome of *B. juncea *(A1–A10; present study) and *B. napus *(N1–N10) [16] and (b) the B genome of *B. juncea *(B1–B8; present study) and *B. nigra *(G1–G8) [18]. Blocks identified in only one of the two studies being compared have been highlighted by a grey background. Based on the non-rearranged blocks that flank the centromere in the AK (ancestral karyotype) and At (*A. thaliana*), the putative centromeric location (represented by solid circles) has been highlighted for the linkage groups wherever possible. New blocks proposed in the present study not shown earlier by Schranz et al. [15] have been marked with an asterisk (*). Blocks with markers from the loci (pericentromeric regions of At) not defined by Schranz et al. [15] have been marked with a question mark (?). Blocks placed within brackets represent insertions within a bigger block.

The A genome of *B. juncea *(present study) was compared with the A genome of *B. napus *[16] based on the arrangement of the 24 genomic blocks. For comparison, all the RFLP markers mapped on *B. napus *were converted to corresponding At loci based on the information available in the supplementary Table S1 of Parkin et al. [16] and assigned to different blocks (A-X) (see Additional file 2). The comparative block arrangement in the A genomes of *B. juncea *and *B. napus *has been shown in Figure 6a.

In terms of the arrangement of the blocks, the A genomes of both *B. juncea *and *B. napus *were essentially collinear. We identified five new blocks (M-N in A1, G-H in A3 and H block in A9) in the A genome of *B. juncea *which were not detected in the corresponding A genome LGs of *B. napus *(Figure 6a). All these new blocks were identified in regions with a reasonably high density of IP markers. Additionally, we also established the presence of some blocks (O-P in A2/N2, N in A3/N3, X and H in A9/N9) (Figure 6a) in the A genome of both *B. juncea *and *B. napus *which were not designated earlier in *B. napus *by Schranz et al. [15]. Block status to these regions were assigned as we observed the presence of At loci from these blocks not only in the homoeologous LGs of the A and C genomes of *B. napus *[16] but also in the corresponding LGs of the A genome of *B. juncea *in the present study (blocks marked with asterisk in Figure 6a). Blocks L, W and X observed in the linkage groups N2, N3 and N6 of *B. napus *(Figure 6a) could not be detected in the homoeologous A genome LGs of *B. juncea *in the present study. This, in all probability, is due to the absence of IP markers representing these blocks in the map. These regions show up as large gaps in the map shown in Figure 1, 2, 3.

Potential centromeric regions were predicted in the linkage groups A3, A4, A5 and A7 of *B. juncea *based on the non-rearranged blocks that flank the centromere in AK and At as proposed by Schranz et al. [15]. The I-J non-rearranged blocks in A4 represent the conserved centromeric regions between the Brassica species and AK, while the G-H non-rearranged blocks in A7 represent the conserved centromeric regions between the Brassica species, AK and At. Similar centromeric regions were predicted for the corresponding N4 and N7 LGs of *B. napus *[16]. In A3 and A5 of *B. juncea*, alternate centromere sites could also be predicted (Figure 6a) due to the presence of mapped markers corresponding to the pericentromeric regions in At. In N3 of *B. napus*, a conserved centromere was predicted between the J-I blocks [15]. However, our mapping data on *B. juncea *suggests that the centromeric region is present between the G-H blocks. All the five markers representing the G-H blocks in A3 were derived from the pericentromeric region of At C2 (Figure 1). Moreover, A3 shared the block arrangement of F-G-H with the ancestral karyotype (AK chromosome 3) [15] unlike in At where the F and G-H blocks are located on different chromosomes. The possibility of a centromere between the J and I blocks in A3 is further weakened by the fact that the orientation of the J block is inverted with respect to the I block in A3 of *B. juncea *which is otherwise non-rearranged in AK [15]. In N5 of *B. napus*, a centromere is predicted between the B-C blocks [15]. Based on the presence of several markers from the pericentromeric region between the J and I blocks in A5 of *B. juncea*, an alternate probable site of the centromere has been predicted in this linkage group.

### Comparative block arrangement in the B genomes of *B. juncea *and *B. nigra *vis-à-vis At

Comparative organization of the A genomes of *B. juncea *and *B. napus *(described above) based on the block boundaries defined by Schranz et al. [15] confirmed that the set of 24 genomic blocks (A-X) can be used to delineate the genomic organization of Brassica genomes. The same block definition was therefore used to study the segmental organization of the B genome of *B. juncea*. The segmental structure of the B genome (B1–B8) of *B. juncea *in relation to the At genome is represented schematically in Figure 4, 5. Since our study allows for a detailed gene-to-gene alignment between the B genome of Brassica and At which has not been reported earlier, the description of the genomic organization of the eight linkage groups (B1–B8) with respect to At is described in detail below.

B1 (G7/J17): B1 predominantly consists of two long stretches of collinear genes from At C3 (block F), one each at the two ends of the linkage group. The two F blocks constitute about 62% of the total mapped area. The colinearity of gene order in comparison with At is suggestive of at least one inversion each in both the F blocks signifying that inversions occurred prior to the diversification of the two blocks. The orientations of the F blocks are inverted with respect to each other. The presence of duplicated F blocks on a single linkage group appears to be a unique feature of the *B. nigra *genome and has not been observed in the A and C genomes. The middle segment of B1 (comprising around 10% of the LG) has markers from block Q (At C5) and block D (At C1).

B2 (G5/J15): This LG consists of four blocks, E-M-R-U, with the E block (At C1) constituting 20% of the top segment and the U block (At C4) constituting 54% of the lower segment of this linkage group. Except for minor rearrangements, the gene order in the U block is highly collinear with its corresponding At C4 region. These rearrangements also explain the presence of markers from the adjacent R and M blocks in this region.

B3 (G8/J18): B3 is constituted of blocks N-M-O-P-J-W-R. Blocks N and M, constituting the top segment of B3, share colinearity of gene order with their counterparts in At. Blocks O and P harbour at least one inversion each which explains the break in colinearity of the gene order as compared with At. The middle segment of B3 is represented by block J (At C2). A minimum of two inversions within the J block could have resulted in the reshuffling of gene markers as compared with At. Blocks R and W constitute the terminal region of B3. A mixture of markers from At C2, At C3 and At C4 is seen between the J and W blocks. Three of these markers viz. At3g47370, At4g07666b and At4g09820 belong to blocks M and P. Their placement between the J and W blocks could be a consequence of the inversions described above.

B4 (G6/J16): B4 consists of small collinear stretches contributed by all the five chromosomes of At. The block order is represented by E-T-N-A-J, with an X block insertion within the J block. The top of the LG is made up by blocks E, T and N. The arrangement of the genes in the N block is suggestive of rearrangements within the block.

B5 (G1/J11): This linkage group consists of two major blocks: F, constituting 30% and J contributing 12% of the total mapped area. The gene order in these two blocks are collinear with the F and J blocks of At.

B6 (G4/J14): About 70% of the total mapped area in the lower part of the B6 linkage group is made up of blocks from At C1 in the order C-B-A-D. The upper part of the linkage group shows the presence of the K block from At C2 while the remaining portions above the K block could not be assigned to any syntenous block as no At loci could be mapped to this region.

B7 (G3/J13): This LG consists predominantly of gene markers from At C1, representing ~53% of the mapped area. The block order is A-B-U-A-E-H. The distribution of the At C1 specific markers is suggestive of the presence of three large collinear regions separated by large gaps and constituting the top, middle and terminal segments of B7. The top portion of this LG comprises of an A block harboring a minimum of one inversion breaking its colinearity with its counterpart in At. The middle segment is formed by a collinear stretch of genes belonging to the B and U blocks. The terminal segment of B7 comprises another A block followed by a long stretch of genes belonging to block E. The gene order in this block indicates two inversions since its divergence from At.

B8 (G2/J12): This is a highly chimeric linkage group comprising nine blocks [A-G-H-I-W-R-U (with an N-Q segment inserted within the U block)] with loci derived from all the five chromosomes of At. The terminal segment of the linkage group is consists of a U block from At C4 with insertions from the N and Q blocks. The gene order of At loci in this block revealed the presence of two sub-blocks ordered in opposite orientations. This break in colinearity can be explained by at least one major inversion which would also explain the appearance of two regions harboring markers from the Q block. The middle portion of this LG is formed of a large collinear region from At C5 (block R-W). Above the R-W block, there is a contiguous stretch of markers derived from blocks G, H and I. This contiguous block arrangement, G-H-I, is observed in At C2 whereas it is shared between linkage groups 3 (G-H) and 4 (I) in AK with a predicted centromere between the G and H blocks in both At and AK [15]. On the basis of the foregoing, a conserved region constituting the centromere could be predicted between the G and H blocks in B8.

Lagercrantz [18] reported a comparative map in *B. nigra *by mapping 284 RFLP loci generated from 160 At DNA fragments. To compare the block arrangement in the B genome of *B. juncea *with its diploid progenitor *B. nigra*, the RFLP loci of the *B. nigra *map, wherever possible, were converted to orthologous At loci and assigned to different blocks (A-X) (see Additional file 3). For the conversion, the RFLP probes whose GenBank sequences could be retrieved, were subjected to NCBI BLASTN search to assign the best corresponding homologous At loci. For the other RFLP probes (which mapped on different At physical maps available at TAIR [31], the corresponding map position in the AGI physical map was determined and the closest At locus name was assigned to the RFLP probe. The comparative block arrangement observed in this study and that reported by Lagercrantz [18] is shown in Figure 6b. Although a general colinearity is observed between the two maps, we identified some new blocks (T in B4 and G, H, I in B8) in the B genome of *B. juncea *which were not detected in the earlier study. Conversely, we also identified some blocks in the *B. nigra *map [18] (C in G1, V in G2, U in G3, Q and V in G4, W in G5 and S in G6) which could not be identified in the B genome of *B. juncea *in this study (Figure 6b and Additional file 3). These discrepancies in block arrangements between the two maps could be due to a limited coverage of At loci in the B genome of *B. juncea *(Table 1) and our inability to convert some of the At RFLP loci described earlier [18] in the *B. nigra *map. Further saturation of the B genome map would facilitate greater accuracy in assigning block status to these regions found to be different between the two genomes. Among the different blocks identified in the B genome, F, J, R and U were the major blocks with the maximum coverage. These were represented as three homoeologous blocks in both the maps. A similar triplication for three of these blocks (F, J and R) was also reported in the previous study by Lagercrantz [18].

### Identification of homoeology between the A, B and C genomes of Brassica species

**Figure 7:**
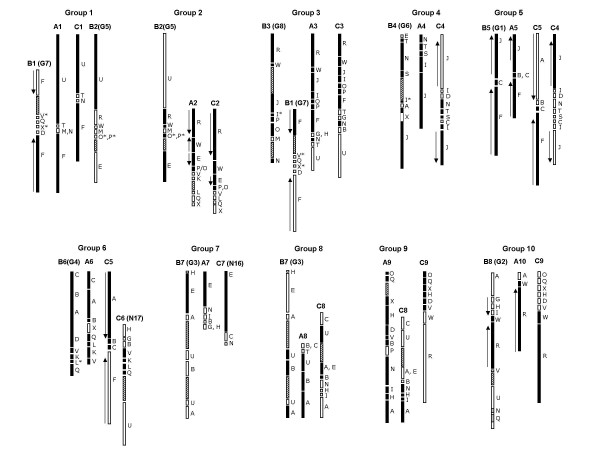
The block arrangements in the A and B genomes are based on the consensus block arrangement of the A genomes of *B. juncea *(A1–A10; present study) and *B. napus *(N1–N10) [16] and the B genomes of *B. juncea *(B1–B8; present study) and *B. nigra *(G1–G8) [18]. The C genome is based on the *B. napus *map (N11–N19) [16]. The original nomenclature of the 2 LGs of the C genome (N16 and N17) and all the LGs of the B genome (G1–G8) have been shown in parentheses with the re-designated nomenclature. Certain single gene insertions were considered as putative blocks (marked with asterisk) if a similar block was found present at the corresponding region in the homoeologous chromosome. Filled bars represent the common blocks shared between all the three members of the group. Large gaps (≥10 cM regions devoid of any markers) in the LGs have been depicted by hatched boxes. Arrows represent the orientation of the gene order (within the block) with respect to the corresponding regions in At.

Considerable homoeology has been reported between the LGs of the A and C genomes [16]. With the mapping information for the B genome derived from the present study, we were able to compare the block-based architecture of the LGs of the three diploid Brassicas to understand the extent of homoeology shared between the three genomes. This analysis provided insights into the prominent rearrangements that shaped the three genomes and also identified those ancestral blocks which remained unaltered in all the three diploid Brassica genomes. For this comparative study, the RFLP loci of the C genome of *B. napus *[16] were converted to their corresponding At loci (Additional file 4). Figure 7 provides an overall view of the homoeology between the three genomes.

The comparative map of the A (A1–A10), B (B1–B8) and C (C1–C9) genomes of Brassica (Figure 7) is based on the consensus block arrangements identified in the comparative map of the A genome of *B. juncea *(A1–A10 of the present study) and *B. napus *(N1–N10) reported earlier [16], B genome of *B. juncea *(B1–B8; present study) and *B. nigra *(G1–G8) reported earlier [18] and C genome of *B. napus *(N11–N18) [16]. This comparative analysis shows that all the linkage groups of the C genome except N16 and N17 [16] correspond to LGs of the A genome based on the extent of the homoeology between them. Hence, we propose that N16 and N17 should be designated as C7 and C6 respectively (Figure 7). Based on the homoeology observed among the A, B and C genomes in our study, a similar nomenclature is proposed for the B genome (B1–B8). This homoeology-based designation will enable greater accuracy and uniformity in the international nomenclature of the three diploid progenitor genomes.

**Figure 8:**
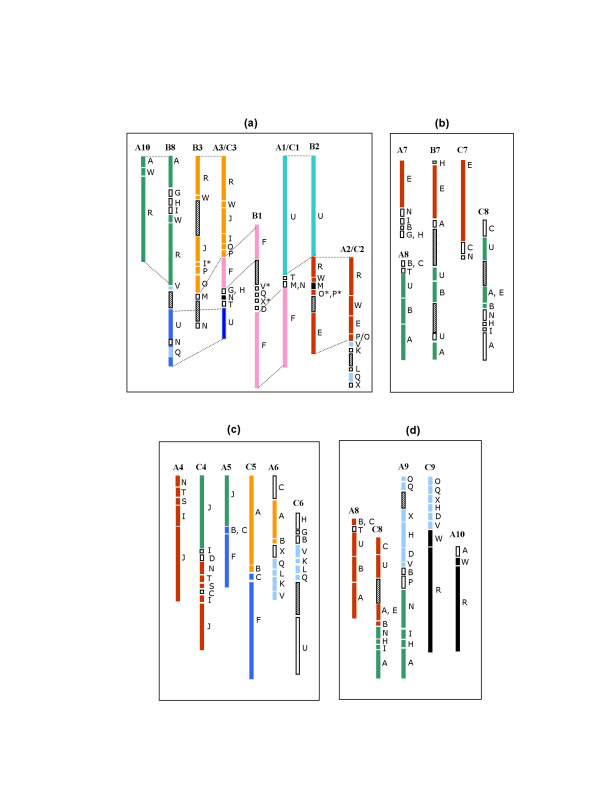
Major rearrangements between the *rapa*/*oleracea *and *nigra *genomes (a, b) and the *rapa *and *oleracea *genomes (c, d). Boxes bearing the same color represent homoeologous blocks while the hatched boxes represent large gaps (≥10 cM regions devoid of any markers). (a) Prominent rearrangements between the A/C and B genomes resulting in altered block arrangement (b) Block organization of one LG of the B genome harbours the blocks from two LGs in the A/C genome explaining the difference of one chromosome between the two genomes (c) *B. oleracea *genome-specific rearrangements (mainly translocations) after the divergence from the Rapa lineage (d) Block organization of two LGs of the C genome (C8, C9) can be derived from three LGs (A8, A9, A10) of the A genome suggesting that the difference of one chromosome between the A and C genomes could be either due to the gain of one of the these LGs in the Rapa lineage or a reduction of one LG in the *oleracea *genome.

All the linkage groups belonging to the three diploid Brassica species could be divided into ten categories or groups (Group1–10) based on the extent of homoeology between them (Figure 7). Group 1 consists of A1/B1–B2/C1. A1 was entirely homoeologous to C1, both being constituted by the block arrangement F-T-U. The F-T-U arrangement was specific to the Rapa/Oleracea lineage. Interestingly, this F-T-U arrangement was found repeated in both the A (A3) and the C (C3) genomes (Group 3, Figure 7) but was absent from the B genome. The linkage group B1 shared the F block with A1/C1 in Group1. Due to the presence of two F blocks in B1, this linkage group also had homoeology with Group 3. B2, which shared the U block with A1 and C1, could also be placed in this group. In Group 2 (A2/B2/C2), A2 and C2 were completely homoeologous, while B2 showed homoeology with A2 and C2 for the block motifs R-W-E-O-P. One inversion in B2 could explain the separation of the E block from the R-W block combination (Figure 8). Group 3 (A3/B1–B3/C3) members shared the common block arrangement R-W-J-I-P-O. B3 was almost entirely composed of this block arrangement, while an additional F-T-U block was present in A3 and C3. Members of Group 4 (A4/B4/C4) shared the block motif J-I-S-N-T. A4 and B4 appeared to be homoeologous along their entire length, while C4 had acquired an additional J block. The presence of two J blocks in C4 was a C genome-specific rearrangement. Due to the presence of an additional J block, C4 has also been placed in group 5. In Group 5 (A5/B5/C4–C5), A5 and B5 were homoeologous along their entire length sharing the block motif J-C-F. C5 shared partial homoeology (blocks F-C) with A5 and B5. The terminal J block present in A5 and B5 was however absent in C5. In Group 6 (A6/B6/C5–C6), A6 and B6 were homoeologous along their entire length sharing the block arrangement C-B-A-V-K-L-Q. One inversion in either of the two genomes could explain the reverse orientation of the blocks constituting the lower segment of A6–B6 (blocks V-K-L-Q). Two C genome LGs (C5 and C6) are also components of this group. C5 shared the A-B-C block arrangement with A6/B6 while C6 was homoeologous for the block arrangement V-K-L-Q with A6/B6. Members of Group 7 (A7/B7/C7) shared homoeology for a large E block while Group 8 (A8/B7/C8) members were homoeologous for blocks A-B-U. Group 9 (A9/C8–C9) had A9 sharing homoeology with C9 for the blocks O-Q-X-H-D-V which constituted the top half of both the linkage groups. The lower segment of A9 shared the block arrangement N-I-H-A with C8. No LG from the B genome seemed to possess any major block homoeologous with A9. Members of group 10 (A10/B8/C9) shared the blocks R-W which constituted a major portion of the linkage group in all the three genomes.

### Karyotype changes that led to the divergence of A, B and C genomes of Brassica species

The identification of homoeologous chromosomes among the three genomes of Brassica sp. (A, B and C) in the present study allows us to predict the possible, macro-level karyotype changes that led to the divergence of the A, B and C genomes. It has been predicted that the Nigra (B) and the Rapa/Oleracea (A/C) lineages separated from each other about 7.9 Mya [32] followed by the splitting of the Rapa and Oleracea lineages. Three linkage groups of the B genome (B4, B5 and B6; Figure 7) have retained similar block organization as their corresponding A genome LGs (A4, A5 and A6; Figure 7). For the remaining five B genome chromosomes (B1, B2, B3, B7 and B8), we propose two types of changes: (1) Rearrangements without any change in the chromosome number: blocks constituting four LGs of the A/C genome (A1–C1/A2–C2/A3–C3/A10) could be reshuffled to explain the block arrangement in four LGs in the B genome (B1/B2/B3/B8) (Figure 8a). No reduction in chromosome number seems to have taken place here. In the process however, both the F-T-U block motifs of the A/C genome lost their identity in the B genome while a unique LG with two F blocks emerged in the B genome. (2) Rearrangements with variations in chromosome number: the genomic block arrangement of B7 could be derived by fusing two linkage groups from the A/C (A7–C7/A8–C8) genome (Figure 8b). This also explains the difference of one LG between the A/C and B genomes.

A high level of similarity between the A and C genomes was established in earlier studies [16]. However, it was not clear whether the changes that were observed between the two occurred in one of the lineages after their divergence or occurred independently in both the lineages. The similar organization of A1–C1, A2–C2, A3–C3 and A7–C7 LGs of the A and C genomes (Figure 7) indicated the absence of any structural changes in these chromosomes after the divergence of the A and C genomes. We therefore predict the following types of changes in the evolution of the A and C genomes: (1) Rearrangements related to the C4, C5 and C6 chromosomes are specific to the C genome (Figure 8c) and occurred after the diversification of the A and C genomes since the corresponding LGs of the A and B genomes are identical (Figure 7). These changes, mainly translocations, break the homoeology between the A and C genomes (Figure 8c). The remaining two LGs (C8 and C9) appear to be made up of rearranged blocks constituting three LGs of the *rapa *genome (A8, A9 and A10; Figure 8d). The block arrangement of C9 can be achieved by fusing half of A9 with almost the entire A10 LG. Similarly, the other half of A9 and the entire A8 represent the block arrangement of the C8 chromosome (Figure 8d). These rearrangements would have contributed to the difference of one chromosome between the A and C genomes.

## Discussion

In this study, we generated a detailed comparative map between *B. juncea *and *Arabidopsis thaliana *containing 533 At loci including 486 IP markers. Although exons of At and Brassica species share around 75–90% homology [33], tapping polymorphism available in the intronic sequences is an efficient method to generate PCR-based markers from otherwise highly conserved genes. Thirty two percent of the primers designed in this study were found to be polymorphic between the *B. juncea *lines, Varuna and Heera, used as parents of the mapping population. Screening the amplified fragments for SNPs could lead to a further increase in the number of polymorphic loci. Previous reports on comparative analysis between At and Brassica species have been based mostly on RFLP probes [16,18-20,24]. Screening large segregating populations with RFLP markers is rather cumbersome. In contrast, the polymorphisms obtained using IP markers were easily discernable on simple 1.5–2.0% agarose gels and would therefore enable rapid screening of large segregating populations. Additionally, being genic in origin, IP marker-based genetic maps would directly reflect the syntenic relationship between the two species, *B. juncea *and At. Since the IP markers used in this study were designed from conserved sequences between At exons and available Brassica EST/GSS sequences, they also possess enormous potential for wider applicability across various Brassica species. As the At genome is partially duplicated [34], the use of multicopy At loci to establish syntenic relationships in other species would raise issues of paralogy and orthology. In contrast, analyzing the distribution of At singletons in Brassica would give an unambiguous representation of various rearrangements that have occurred between the two lineages since their divergence. The major emphasis in this study was therefore on generating IP PCR primers predominantly from single copy loci in Arabidopsis. Our genomic block definitions were based mostly on At singletons in contrast to earlier studies [16]. Irrespective of the approach used to define synteny, the number of times each block is represented in the A genome of *B. juncea *was found to be highly similar to that observed for *B. napus*. This observation supports the earlier hypothesis which advocated the occurrence of duplications in the Arabidopsis genome prior to its divergence from the Brassica lineage [16].

In the present study, we analyzed the segmental organization of the *B. juncea *genome based on the 24 conserved genomic blocks described by Schranz et al. [15]. These blocks represent the conserved regions common to the AK, At and *B. rapa *[15]. Block order obtained for *B. juncea *in this study was compared with the available maps for the A and C constituent genomes of *B. napus *[16] and the B genome of *B. nigra *[18]. A high level of conserved macro-level colinearity was observed between *B. juncea *and its diploid progenitors. The *rapa *(A) genomes of both *B. juncea *and *B. napus *were found to be highly comparable (Figure 6a). Similarly, the B genome of *B. juncea *appeared to maintain similar genomic block architecture as its diploid counterpart in *B. nigra *(Figure 6b). This signified the absence of large scale perturbations during the formation of the allopolyploid Brassicas contrary to earlier reports by many groups working on synthetic Brassica polyploids [35-37]. Additionally, the conserved identity of both the constituent diploid genomes in the Brassica polyploids suggests the involvement of a strong and active gene action which inhibits pairing between the homoeologous chromosomes. Thus, survival of the allopolypoids *B. napus*, *B. juncea *and *B. carinata*, in all probability, was based on hybrid vigour and suppression of pairing of the homoeologous chromosomes.

Defining the constitution of the A, B and C Brassica genomes based on these genomic blocks facilitated the reconstruction of the ancestral karyotype to decipher the evolution and diversification of the Brassica lineage. Common block arrangements shared between the three lineages suggest an ancestral origin. Several such conserved block motifs were identified in this study. These include blocks R-W-E-O-P (Group 2); blocks R-W-J-I-O-P (Group 3) and blocks J-I-S-T-N (Group 4) (Figure 7). Since the two lineages within the tribe Brassiceae (Nigra lineage and Rapa/Oleracea lineage) diverged approximately ~7.9 Mya [32], the block motifs shared by the A and B genomes would also be ancestral. Thus, the block motifs shared between the members of Group 5 (blocks J-C-F) and Group 6 (blocks C-B-A-V-K-L-Q) (Figure 7) are speculated to be ancestral in origin although their identity in the Oleracea lineage is lost. Based on the comparative genome analysis of the three diploid Brassica species carried out in this study the constitution of the ancestral Brassica karyotype (ABK) in terms of the block arrangement could be predicted for at least five LGs (ABK2–ABK6; see Additional File 5). Earlier studies on chloroplast DNA analysis [38,39] clearly established that the *B. rapa*/*B. oleracea *are very closely placed in one lineage and *B. nigra *belongs to a different lineage. Our results suggest significant similarity between the A and B genome for five linkage groups. We therefore propose that besides the five LG putative ancestor, there were divergent parents (female) involved in the evolution of the two major Brassica lineages.

Comparative analysis of block organization revealed certain major rearrangements (translocations and fusions) that were pivotal to karyotype diversification in the different Brassica lineages. Complete homoeology in terms of block organization was seen for three linkage groups each between the *rapa *(A) and *oleracea *(C) genomes (A1–C1, A2–C2 and A3–C3; Figure 7) and the *rapa *(A) and *nigra *(B) genomes (A4–B4, A5–B5 and A6–B6; Figure 7). This implies that although the Rapa and Oleracea lineages are more closely related, certain block arrangements have been retained by the Rapa and Nigra lineages while being lost from the C genome. If the rearrangements in the block organization of the remaining LGs are compared, rearrangements leading to lineage-specific karyotype diversification become evident. Our study also highlights certain rearrangements (Figure 8c) that appear to have originated in the Oleracea lineage after its divergence from Rapa. Since the chromosome number of the common Brassica ancestor remains uncertain, one cannot discern whether the splitting of chromosomes led to the successive increase in chromosome number in the three Brassica lineages or fusions led to a reduction in the chromosome number. However, in the present study, the rearrangements leading to the difference of one LG each between the A-B and A-C lineages could be deciphered (Figure 8b, 8d).

Previous studies suggested that two reciprocal translocations, three chromosome fusions and at least three inversions were instrumental in the derivation of At from the ancestral karyotype-AK [40,41]. In the process, several new block boundaries were created. We compared the genomic block organization of AK, At and the three Brassica genomes to identify new/common block junctions to gain further insight into the evolution of the crucifer genomes. In the three Brassica genomes analyzed, block R was found to be almost always associated with block W (Figure 7) and block Q was associated with block X. These associations are characteristic of the Brassica genome and are not found in AK or At [15]. The *rapa/oleracea *A3/C3 LGs shared the ancestral pattern of blocks F-G-H (AK chromosome 3) [15] while in At, F and G-H occupy different chromosomes. Similarly, with the extent of saturation available for the three genetic maps of Brassica, the C-D, K-G and Q-S block fusions characteristic of At are lacking in Brassica. This suggests that different rearrangements led to the formation of the Arabidopsis and the Brassica ancestor. A more detailed view of karyotype evolution would be available once all the centromeres for the Brassica genomes are identified.

The identification of homoeology based on the conserved 24 genomic blocks would help build a unified comparative genomics system in the Brassicaceae as has been done for the Gramineae family by the generation of the 'Crop-Circle' [42-44]. This will facilitate the transfer of information from one species to another for which IP markers would be most useful. With the elucidation of genes involved in many biochemical pathways and the availability of gene expression data, information generated in the model species Arabidopsis holds enormous potential for application in breeding of Brassica crops. Establishment of syntenic relationships between At and Brassica would be highly beneficial for the identification of candidate genes contributing to traits of agronomic value from corresponding regions in At and also serve as an exhaustive resource to generate more markers for fine mapping in syntenic regions of Brassicas.

## Conclusion

The present study conclusively establishes the efficacy of IP markers for the development of a comparative map between a model plant and a polyploid crop species. The comparative genome analysis performed in this study has contributed significantly to our understanding of the homoeology shared between the A, B and C Brassica genomes. The study also identifies, at the macro-level, the evolutionary rearrangements leading to karyotype diversification among the three genomes. Additionally, some of the putative ancestral Brassica-specific block motifs were identified. Syntenic relationships thus established between *B. juncea*, *B. napus*, *B. nigra *and At will facilitate precision breeding and identification and positional cloning of candidate genes contributing to traits of agronomic value in Brassica species.

## Methods

A total of 1180 primer pairs (247 from At C1, 193 from At C2, 214 from At C3, 292 from At C4 and 234 from At C5) were designed. Genes having larger introns were preferred as the incidence of indels is expected to be higher in such cases. The expected amplicon size for the designed primer pairs was approx. 500 bp-1 kb in *A. thaliana*. To reduce non-specific amplifications in *B. juncea*, primer pairs designed from At singletons were first tested on At DNA to optimize conditions for PCR which result in a single amplicon. For most of the primer pairs screened on At DNA, the optimum PCR condition was: initial denaturation at 94°C for 5 min, followed by 30 cycles of denaturation at 94°C for 30 s, annealing at 55°C for 30 s and elongation at 72°C for 1 min followed by a final extension at 72°C for 5 min. Amplified PCR products were analyzed on 1.2–2% w/v agarose gel to score for polymorphism either on the basis of size/length difference or presence/absence (dominant) of PCR products. A mapping population consisting of 123 doubled haploid lines derived from a cross between Varuna (an Indian cultivar) and Heera (an east European line) was used for the construction of the linkage map [30]. IP markers were added to a framework map of *B. juncea *described earlier by Pradhan et al. [30] and Ramchairy et al. [11] using the program Joinmap version 2.0 [45,46].

## Abbreviations

At: *Arabidopsis thaliana;* At C1–C5: Chromosomes of At (1–5); AK: Ancestral karyotype; cM: centiMorgan; IP: Intron polymorphism; LG: Linkage group.

## Authors' contributions

PP, AJ, NCB, LP and SS designed intron spanning primers from one each of the five At chromosomes and mapped the IP markers on the F1DH population. PP wrote the paper and put together the figures. The comparative analysis of the A, B and C genomes of Brassica were done by PP. VG contributed to some of the mapping experiments and organized the laboratory work. AKP looked at all the mapping data and helped in organizing the mapping information and the manuscript. DP conceived the project and contributed to the writing of the manuscript. All authors read and approved the final manuscript.

## Supplementary Material

Additional file 1 Sequence data of the intron spanning primers used in the present study. This file contains the sequence information of all the intron spanning primers which generated polymorphic IP markers deployed to develop the *B. juncea *genetic map in the present study. Click here for file

Additional file 2 Comparative genome organization of the A genome of *B. juncea *(A1–A10; present study) and *B. napus *(N1–N10) [16]. This file contains the map of the A genome of *B. napus *[16] with the RFLP loci converted to their corresponding At (*A. thaliana*) loci and a detailed comparison (in terms of the At loci arrangement) of this map with the A genome of *B. juncea *(present study). Click here for file

Additional file 3 Comparative genome organization of the B genome of *B. juncea *(B1–B8; present study) and *B. nigra *(G1–G8) [18]. This file contains the map of the B genome of *B. nigra *[18] with the RFLP loci converted to their corresponding At (*A. thaliana*) loci and a detailed comparison (in terms of the At loci arrangement) of this map with the B genome of *B. juncea *(present study). Click here for file

Additional file 4 The C genome map of *B. napus *[16] with the RFLP probes converted to their corresponding At loci. Click here for file

Additional file 5 Putative ancestral Brassica karyotype (ABK2–ABK6). The file contains the figurative representation of the putative ancestral Brassica karyotype (ABK2–ABK6) predicted in our study. This is based on the conserved group structure of groups 2, 3, 4, 5 and 6 (Figure 7). Only partial organization of the putative linkage groups ABK2 and ABK3 is shown representing the common blocks shared between the three diploid Brassica genomes. Click here for file
