# Supplementary material for: Comparative mapping of Brassica juncea and Arabidopsis thaliana using Intron Polymorphism (IP) markers: homoeologous relationships, diversification and evolution of the A, B and C Brassica genomes
Source: BMC Genomics. 2008 Mar 3;9:113. doi: 10.1186/1471-2164-9-113 (PMC2277410; doi:10.1186/1471-2164-9-113)
Supplement: Additional file 2 — Comparative genome organization of the A genome of B. juncea (A1–A10; present study) and B. napus (N1–N10) [16]. This file contains the map of the A genome of B. napus [16] with the RFLP loci converted to their corresponding At (A. thaliana) loci and a detailed comparison (in terms of the At loci arrangement) of this map with the A genome of B. juncea (present study). [file 1471-2164-9-113-S2.ppt]

## Slide 1
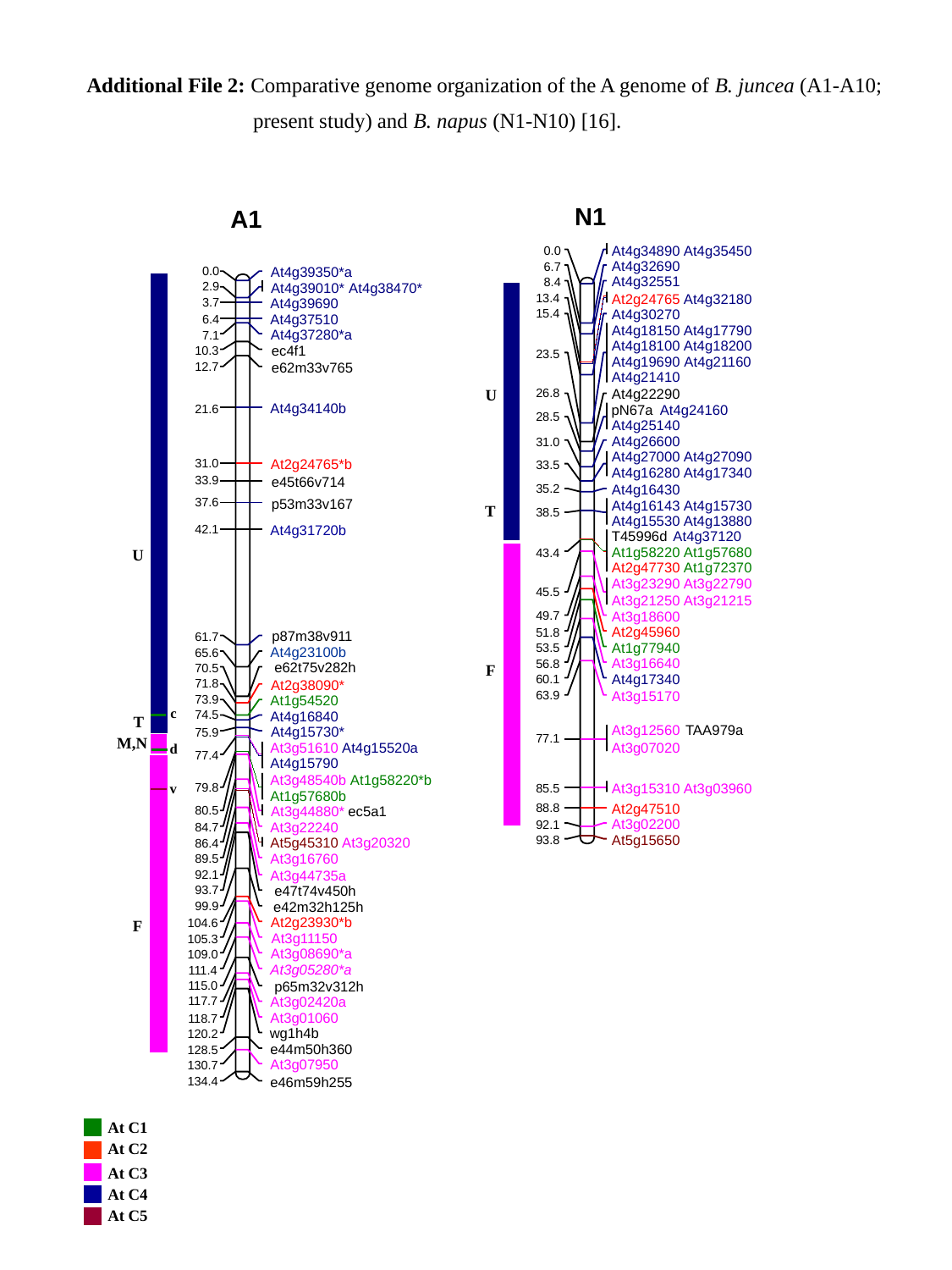

Additional File 2: Comparative genome organization of the A genome of B. juncea (A1-A10; present study) and B. napus (N1-N10) [16].
N1
At4g34890
At4g35450
0.0
At4g32690
6.7
At4g32551
8.4
13.4
At2g24765
At4g32180
15.4
At4g30270
At4g18150
At4g17790
At4g18100
At4g18200
23.5
At4g19690
At4g21160
At4g21410
26.8
At4g22290
pN67a
At4g24160
28.5
At4g25140
At4g26600
31.0
At4g27000
At4g27090
33.5
At4g16280
At4g17340
35.2
At4g16430
At4g16143
At4g15730
38.5
At4g15530
At4g13880
T45996d
At4g37120
At1g58220
At1g57680
43.4
At2g47730
At1g72370
At3g23290
At3g22790
45.5
At3g21250
At3g21215
49.7
At3g18600
At2g45960
51.8
At1g77940
53.5
At3g16640
56.8
At4g17340
60.1
63.9
At3g15170
At3g12560
TAA979a
77.1
At3g07020
At3g15310
At3g03960
85.5
88.8
At2g47510
At3g02200
92.1
At5g15650
93.8
U
F
T
A1
0.0
At4g39350*a
U
c
T
M,N
d
v
F
2.9
At4g39010*
At4g38470*
3.7
At4g39690
At4g37510
6.4
At4g37280*a
7.1
ec4f1
10.3
12.7
e62m33v765
At4g34140b
21.6
31.0
At2g24765*b
33.9
e45t66v714
37.6
p53m33v167
42.1
At4g31720b
p87m38v911
61.7
At4g23100b
65.6
e62t75v282h
70.5
71.8
At2g38090*
73.9
At1g54520
74.5
At4g16840
At4g15730*
75.9
At3g51610
At4g15520a
77.4
At4g15790
At3g48540b
At1g58220*b
79.8
At1g57680b
80.5
At3g44880*
ec5a1
At3g22240
84.7
At5g45310
At3g20320
86.4
At3g16760
89.5
92.1
At3g44735a
93.7
e47t74v450h
99.9
e42m32h125h
At2g23930*b
104.6
At3g11150
105.3
At3g08690*a
109.0
At3g05280*a
111.4
115.0
p65m32v312h
117.7
At3g02420a
At3g01060
118.7
wg1h4b
120.2
e44m50h360
128.5
At3g07950
130.7
134.4
e46m59h255
At C1
At C2
At C3
At C4
At C5

## Slide 2
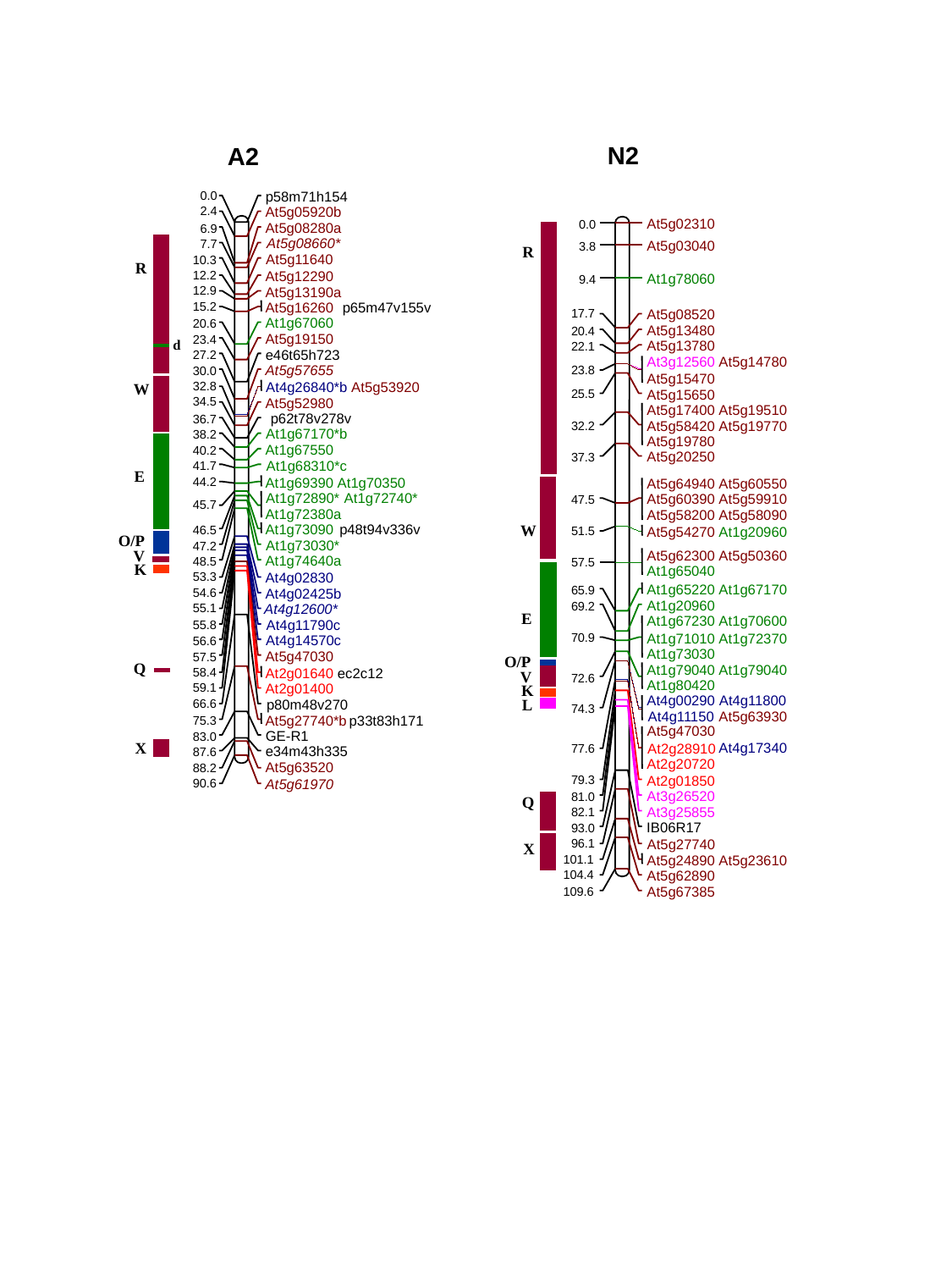

N2
At5g02310
0.0
At5g03040
3.8
At1g78060
9.4
17.7
At5g08520
At5g13480
20.4
At5g13780
22.1
At3g12560
At5g14780
23.8
At5g15470
25.5
At5g15650
At5g17400
At5g19510
At5g58420
At5g19770
32.2
At5g19780
At5g20250
37.3
At5g64940
At5g60550
At5g60390
At5g59910
47.5
At5g58200
At5g58090
51.5
At5g54270
At1g20960
At5g62300
At5g50360
57.5
At1g65040
At1g65220
At1g67170
65.9
At1g20960
69.2
At1g67230
At1g70600
70.9
At1g71010
At1g72370
At1g73030
At1g79040
At1g79040
72.6
At1g80420
At4g00290
At4g11800
74.3
At4g11150
At5g63930
At5g47030
At4g17340
At2g28910
77.6
At2g20720
79.3
At2g01850
At3g26520
81.0
At3g25855
82.1
IB06R17
93.0
96.1
At5g27740
101.1
At5g24890
At5g23610
104.4
At5g62890
At5g67385
109.6
A2
0.0
p58m71h154
2.4
At5g05920b
At5g08280a
6.9
At5g08660*
7.7
At5g11640
10.3
12.2
At5g12290
12.9
At5g13190a
15.2
At5g16260
p65m47v155v
At1g67060
20.6
At5g19150
23.4
e46t65h723
27.2
At5g57655
30.0
32.8
At4g26840*b
At5g53920
34.5
At5g52980
p62t78v278v
36.7
At1g67170*b
38.2
At1g67550
40.2
At1g68310*c
41.7
44.2
At1g69390
At1g70350
At1g72890*
At1g72740*
45.7
At1g72380a
At1g73090
p48t94v336v
46.5
At1g73030*
47.2
At1g74640a
48.5
53.3
At4g02830
54.6
At4g02425b
55.1
At4g12600*
At4g11790c
55.8
At4g14570c
56.6
At5g47030
57.5
58.4
At2g01640
ec2c12
59.1
At2g01400
66.6
p80m48v270
At5g27740*b
p33t83h171
75.3
GE-R1
83.0
e34m43h335
87.6
At5g63520
88.2
90.6
At5g61970
R
d
W
E
V
K
Q
X
O/P
R
W
E
O/P
V
K
Q
X
L

## Slide 3
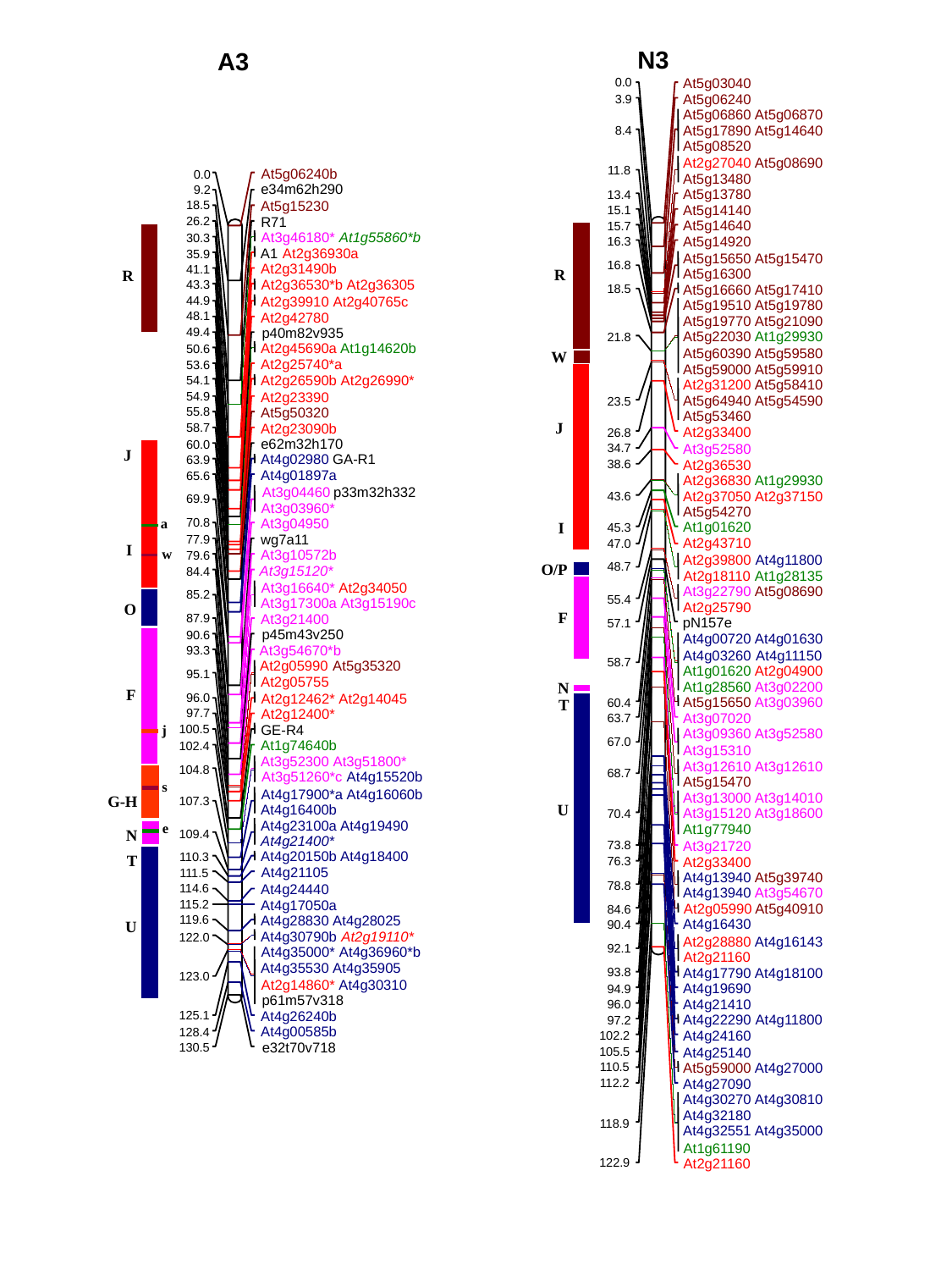

N3
A3
0.0
At5g03040
At5g06240
3.9
At5g06860
At5g06870
At5g17890
At5g14640
8.4
At5g08520
At2g27040
At5g08690
11.8
At5g13480
At5g13780
13.4
At5g14140
15.1
At5g14640
15.7
At5g14920
16.3
At5g15650
At5g15470
16.8
At5g16300
18.5
At5g16660
At5g17410
At5g19510
At5g19780
At5g19770
At5g21090
At5g22030
At1g29930
21.8
At5g60390
At5g59580
At5g59000
At5g59910
At2g31200
At5g58410
At5g64940
At5g54590
23.5
At5g53460
At2g33400
26.8
34.7
At3g52580
38.6
At2g36530
At2g36830
At1g29930
At2g37050
At2g37150
43.6
At5g54270
At1g01620
45.3
At2g43710
47.0
At2g39800
At4g11800
48.7
At2g18110
At1g28135
At3g22790
At5g08690
55.4
At2g25790
pN157e
57.1
At4g00720
At4g01630
At4g03260
At4g11150
58.7
At1g01620
At2g04900
At1g28560
At3g02200
At5g15650
At3g03960
60.4
At3g07020
63.7
At3g09360
At3g52580
67.0
At3g15310
At3g12610
At3g12610
68.7
At5g15470
At3g13000
At3g14010
At3g15120
At3g18600
70.4
At1g77940
73.8
At3g21720
76.3
At2g33400
At4g13940
At5g39740
78.8
At4g13940
At3g54670
At2g05990
At5g40910
84.6
At4g16430
90.4
At2g28880
At4g16143
92.1
At2g21160
93.8
At4g17790
At4g18100
At4g19690
94.9
At4g21410
96.0
At4g22290
At4g11800
97.2
At4g24160
102.2
105.5
At4g25140
110.5
At5g59000
At4g27000
112.2
At4g27090
At4g30270
At4g30810
At4g32180
118.9
At4g32551
At4g35000
At1g61190
122.9
At2g21160
At5g06240b
0.0
e34m62h290
9.2
18.5
At5g15230
26.2
R71
R
J
I
O/P
F
N
T
U
W
R
J
a
I
w
O
F
j
s
G-H
e
N
T
U
At3g46180*
At1g55860*b
30.3
A1
At2g36930a
35.9
At2g31490b
41.1
At2g36530*b
At2g36305
43.3
44.9
At2g39910
At2g40765c
48.1
At2g42780
49.4
p40m82v935
At2g45690a
At1g14620b
50.6
At2g25740*a
53.6
At2g26590b
At2g26990*
54.1
54.9
At2g23390
55.8
At5g50320
58.7
At2g23090b
e62m32h170
60.0
At4g02980
GA-R1
63.9
At4g01897a
65.6
At3g04460
p33m32h332
69.9
At3g03960*
70.8
At3g04950
wg7a11
77.9
At3g10572b
79.6
At3g15120*
84.4
At3g16640*
At2g34050
85.2
At3g17300a
At3g15190c
87.9
At3g21400
p45m43v250
90.6
At3g54670*b
93.3
At2g05990
At5g35320
95.1
At2g05755
96.0
At2g12462*
At2g14045
97.7
At2g12400*
100.5
GE-R4
At1g74640b
102.4
At3g52300
At3g51800*
104.8
At3g51260*c
At4g15520b
At4g17900*a
At4g16060b
107.3
At4g16400b
At4g23100a
At4g19490
109.4
At4g21400*
At4g20150b
At4g18400
110.3
At4g21105
111.5
114.6
At4g24440
115.2
At4g17050a
119.6
At4g28830
At4g28025
At4g30790b
At2g19110*
122.0
At4g35000*
At4g36960*b
At4g35530
At4g35905
123.0
At2g14860*
At4g30310
p61m57v318
125.1
At4g26240b
At4g00585b
128.4
e32t70v718
130.5

## Slide 4
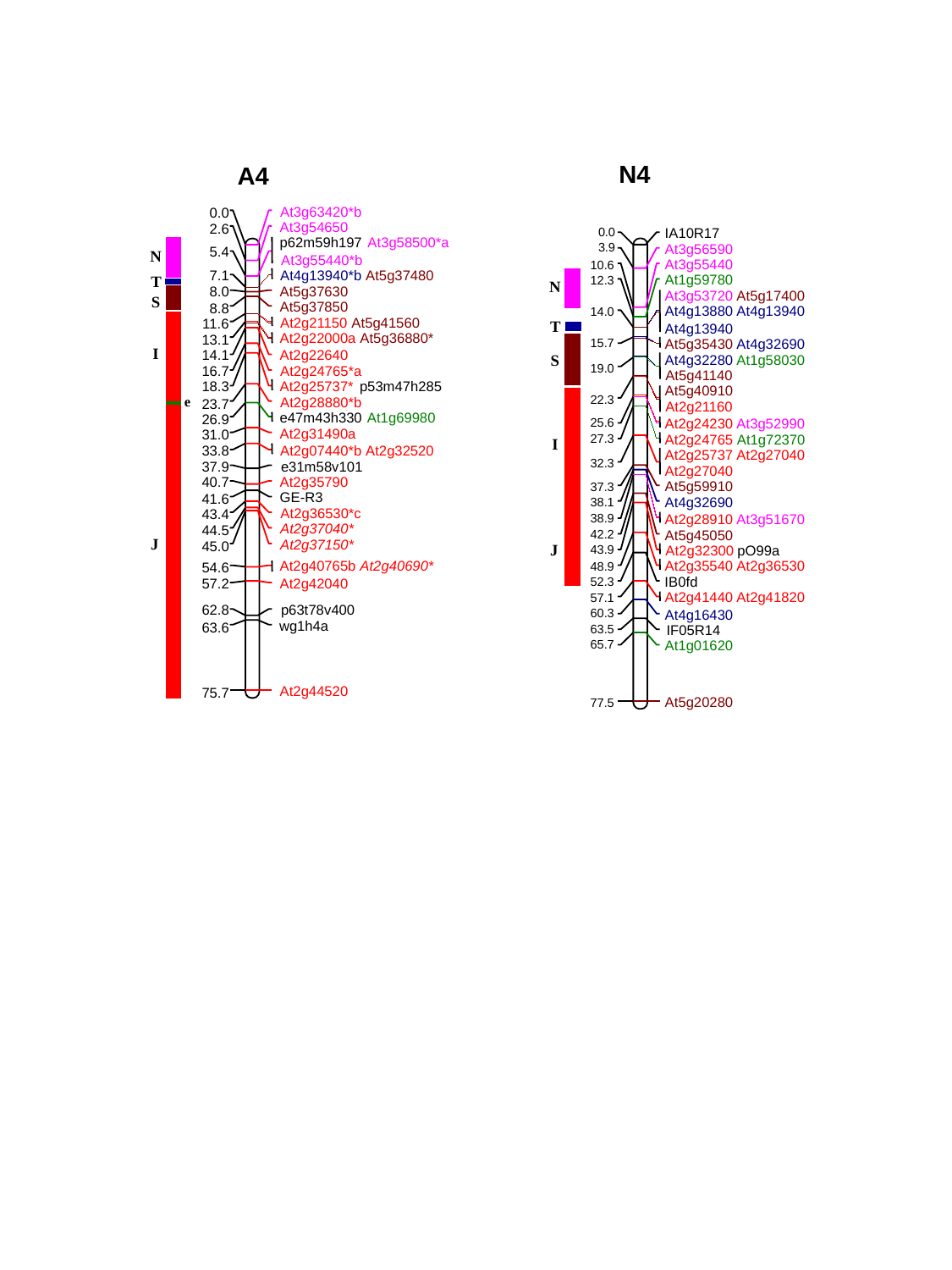

N4
0.0
IA10R17
3.9
At3g56590
At3g55440
10.6
At1g59780
12.3
At3g53720
At5g17400
At4g13880
At4g13940
14.0
At4g13940
15.7
At5g35430
At4g32690
At4g32280
At1g58030
19.0
At5g41140
At5g40910
22.3
At2g21160
25.6
At2g24230
At3g52990
27.3
At2g24765
At1g72370
At2g25737
At2g27040
32.3
At2g27040
At5g59910
37.3
At4g32690
38.1
38.9
At2g28910
At3g51670
42.2
At5g45050
43.9
At2g32300
pO99a
At2g35540
At2g36530
48.9
IB0fd
52.3
At2g41440
At2g41820
57.1
60.3
At4g16430
63.5
IF05R14
65.7
At1g01620
At5g20280
77.5
N
T
S
I
J
A4
At3g63420*b
0.0
At3g54650
2.6
p62m59h197
At3g58500*a
N
T
S
I
e
J
5.4
At3g55440*b
7.1
At4g13940*b
At5g37480
8.0
At5g37630
At5g37850
8.8
At2g21150
At5g41560
11.6
At2g22000a
At5g36880*
13.1
14.1
At2g22640
16.7
At2g24765*a
18.3
At2g25737*
p53m47h285
At2g28880*b
23.7
e47m43h330
At1g69980
26.9
At2g31490a
31.0
33.8
At2g07440*b
At2g32520
37.9
e31m58v101
40.7
At2g35790
GE-R3
41.6
At2g36530*c
43.4
At2g37040*
44.5
At2g37150*
45.0
At2g40765b
At2g40690*
54.6
57.2
At2g42040
62.8
p63t78v400
wg1h4a
63.6
At2g44520
75.7

## Slide 5
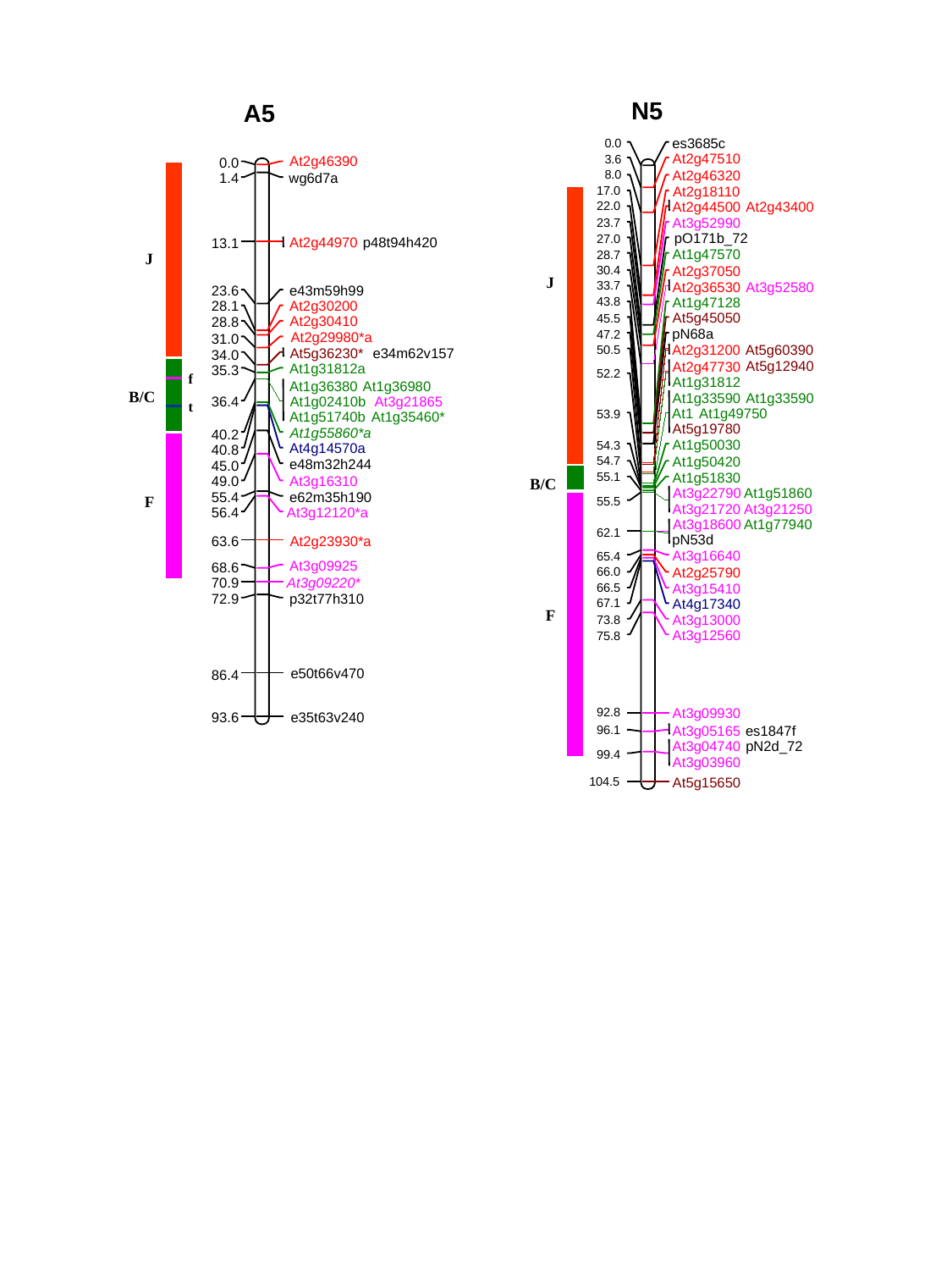

N5
es3685c
0.0
At2g47510
3.6
8.0
At2g46320
17.0
At2g18110
J
B/C
F
22.0
At2g44500
At2g43400
At3g52990
23.7
pO171b_72
27.0
At1g47570
28.7
30.4
At2g37050
33.7
At2g36530
At3g52580
43.8
At1g47128
At5g45050
45.5
pN68a
47.2
At2g31200
At5g60390
50.5
At5g12940
At2g47730
52.2
At1g31812
At1g33590
At1g33590
At1
At1g49750
53.9
At5g19780
At1g50030
54.3
54.7
At1g50420
55.1
At1g51830
At3g22790
At1g51860
55.5
At3g21720
At3g21250
At3g18600
At1g77940
62.1
pN53d
At3g16640
65.4
66.0
At2g25790
66.5
At3g15410
67.1
At4g17340
At3g13000
73.8
At3g12560
75.8
92.8
At3g09930
96.1
At3g05165
es1847f
At3g04740
pN2d_72
99.4
At3g03960
104.5
At5g15650
A5
At2g46390
0.0
1.4
wg6d7a
At2g44970
p48t94h420
13.1
J
23.6
e43m59h99
28.1
At2g30200
At2g30410
28.8
At2g29980*a
31.0
e34m62v157
At5g36230*
34.0
At1g31812a
35.3
f
At1g36380
At1g36980
B/C
t
36.4
At1g02410b
At3g21865
At1g51740b
At1g35460*
At1g55860*a
40.2
At4g14570a
40.8
e48m32h244
45.0
49.0
At3g16310
F
55.4
e62m35h190
56.4
At3g12120*a
63.6
At2g23930*a
At3g09925
68.6
70.9
At3g09220*
72.9
p32t77h310
e50t66v470
86.4
93.6
e35t63v240

## Slide 6
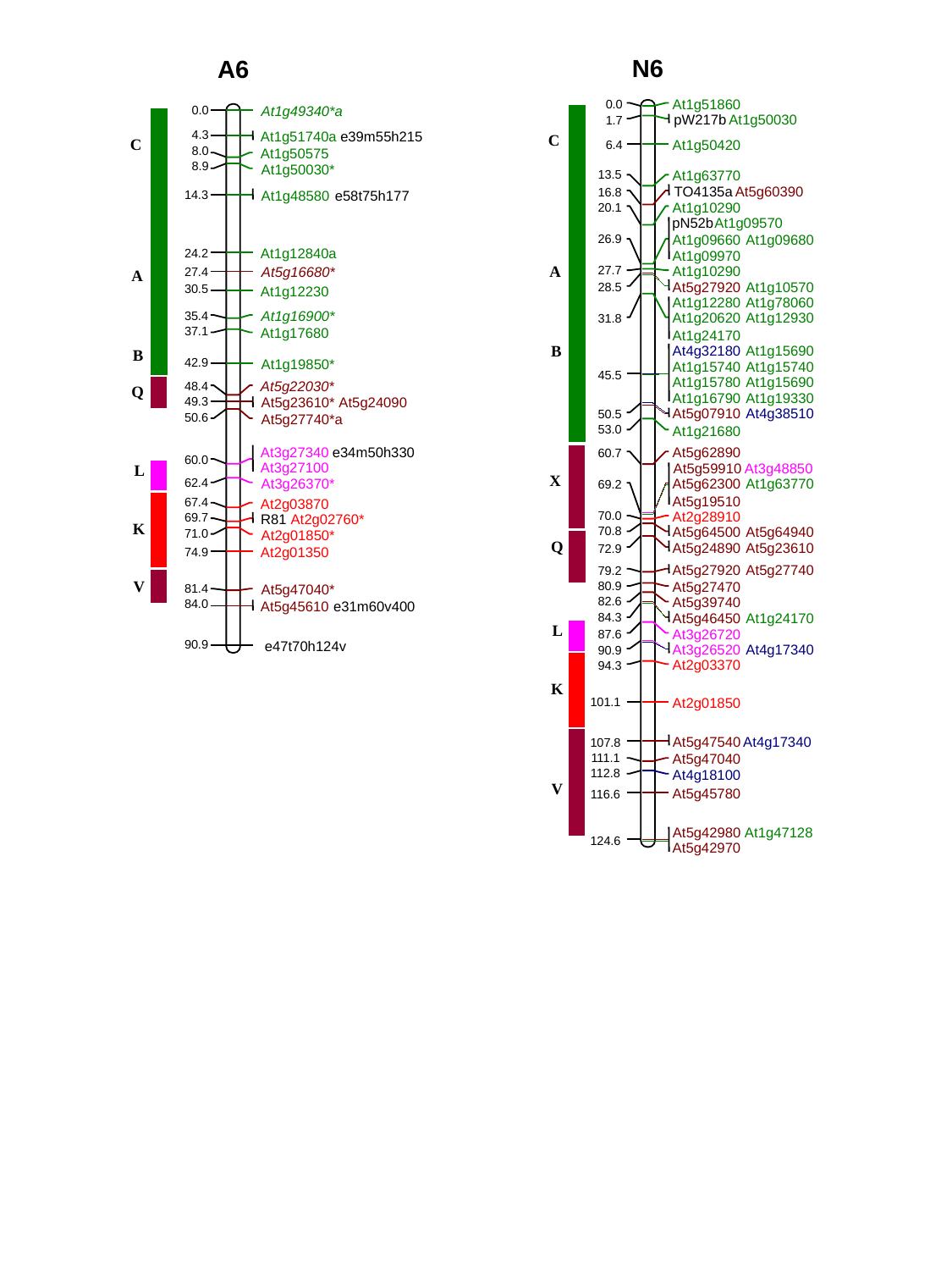

N6
At1g51860
0.0
C
A
B
X
Q
L
K
V
pW217b
At1g50030
1.7
At1g50420
6.4
13.5
At1g63770
TO4135a
At5g60390
16.8
At1g10290
20.1
pN52b
At1g09570
26.9
At1g09660
At1g09680
At1g09970
27.7
At1g10290
At5g27920
At1g10570
28.5
At1g12280
At1g78060
At1g20620
At1g12930
31.8
At1g24170
At4g32180
At1g15690
At1g15740
At1g15740
45.5
At1g15780
At1g15690
At1g16790
At1g19330
At5g07910
At4g38510
50.5
53.0
At1g21680
At5g62890
60.7
At5g59910
At3g48850
At5g62300
At1g63770
69.2
At5g19510
70.0
At2g28910
70.8
At5g64500
At5g64940
At5g24890
At5g23610
72.9
At5g27920
At5g27740
79.2
80.9
At5g27470
82.6
At5g39740
84.3
At5g46450
At1g24170
At3g26720
87.6
At3g26520
At4g17340
90.9
At2g03370
94.3
101.1
At2g01850
At5g47540
At4g17340
107.8
111.1
At5g47040
112.8
At4g18100
At5g45780
116.6
At5g42980
At1g47128
124.6
At5g42970
A6
0.0
At1g49340*a
4.3
At1g51740a
e39m55h215
C
8.0
At1g50575
8.9
At1g50030*
14.3
At1g48580
e58t75h177
At1g12840a
24.2
A
At5g16680*
27.4
30.5
At1g12230
At1g16900*
35.4
37.1
At1g17680
B
42.9
At1g19850*
Q
At5g22030*
48.4
At5g23610*
At5g24090
49.3
50.6
At5g27740*a
At3g27340
e34m50h330
60.0
L
At3g27100
At3g26370*
62.4
67.4
At2g03870
69.7
R81
At2g02760*
K
71.0
At2g01850*
At2g01350
74.9
V
81.4
At5g47040*
84.0
At5g45610
e31m60v400
90.9
e47t70h124v

## Slide 7
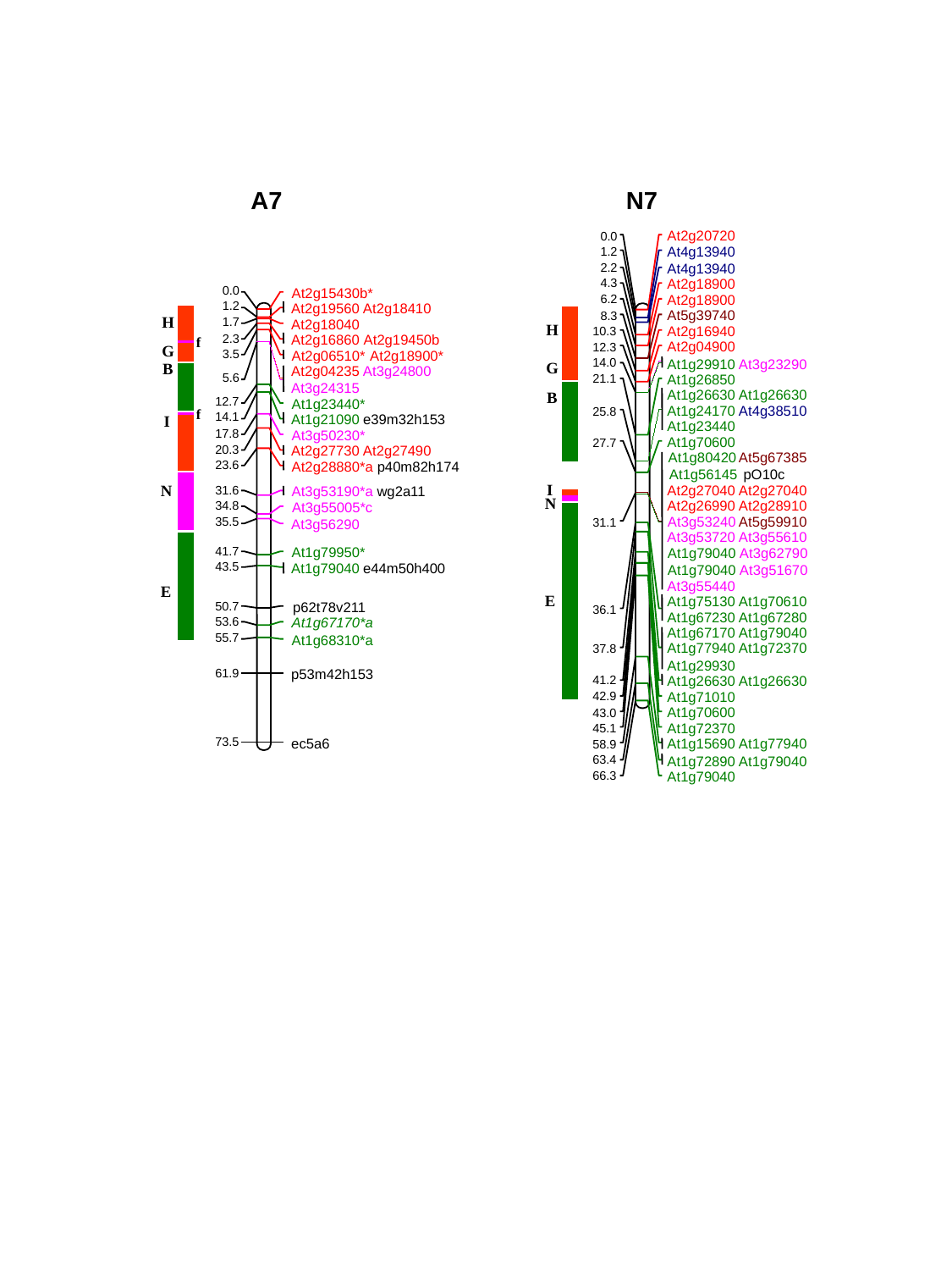

A7
0.0
At2g15430b*
1.2
At2g19560
At2g18410
1.7
At2g18040
2.3
At2g16860
At2g19450b
3.5
At2g06510*
At2g18900*
At2g04235
At3g24800
5.6
At3g24315
12.7
At1g23440*
14.1
At1g21090
e39m32h153
17.8
At3g50230*
20.3
At2g27730
At2g27490
23.6
At2g28880*a
p40m82h174
31.6
At3g53190*a
wg2a11
34.8
At3g55005*c
35.5
At3g56290
41.7
At1g79950*
43.5
At1g79040
e44m50h400
50.7
p62t78v211
53.6
At1g67170*a
55.7
At1g68310*a
61.9
p53m42h153
73.5
ec5a6
H
G
f
B
f
I
N
E
N7
At2g20720
0.0
At4g13940
1.2
2.2
At4g13940
4.3
At2g18900
6.2
At2g18900
At5g39740
8.3
At2g16940
10.3
At2g04900
12.3
14.0
At1g29910
At3g23290
21.1
At1g26850
At1g26630
At1g26630
At1g24170
At4g38510
25.8
At1g23440
At1g70600
27.7
At1g80420
At5g67385
At1g56145
pO10c
At2g27040
At2g27040
At2g26990
At2g28910
At3g53240
At5g59910
31.1
At3g53720
At3g55610
At1g79040
At3g62790
At1g79040
At3g51670
At3g55440
At1g75130
At1g70610
36.1
At1g67230
At1g67280
At1g67170
At1g79040
At1g77940
At1g72370
37.8
At1g29930
41.2
At1g26630
At1g26630
42.9
At1g71010
At1g70600
43.0
At1g72370
45.1
At1g15690
At1g77940
58.9
63.4
At1g72890
At1g79040
66.3
At1g79040
H
G
B
I
N
E

## Slide 8
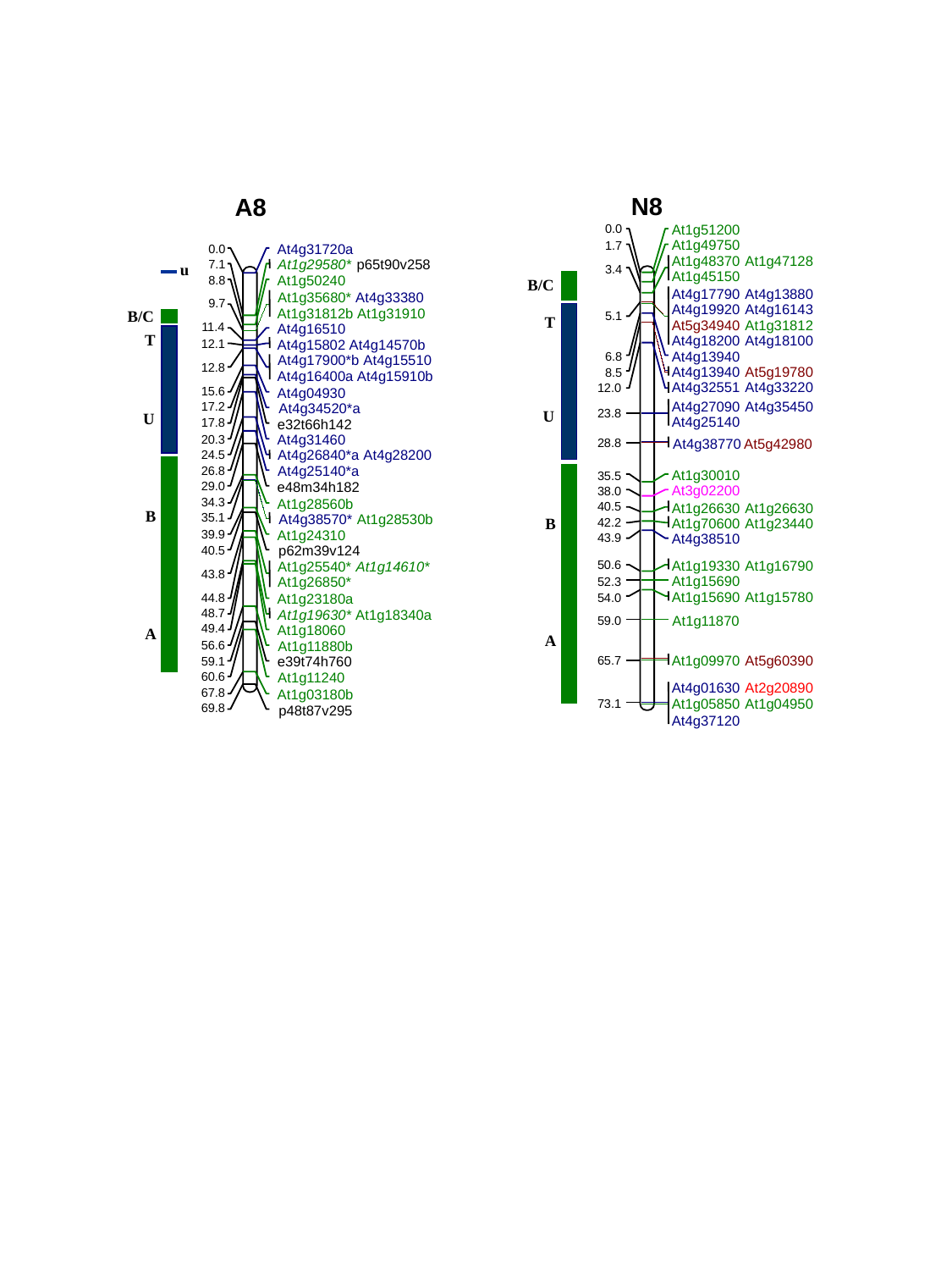

N8
0.0
At1g51200
At1g49750
1.7
At1g48370
At1g47128
3.4
At1g45150
At4g17790
At4g13880
At4g19920
At4g16143
5.1
At5g34940
At1g31812
At4g18200
At4g18100
At4g13940
6.8
At4g13940
At5g19780
8.5
At4g32551
At4g33220
12.0
At4g27090
At4g35450
23.8
At4g25140
28.8
At4g38770
At5g42980
At1g30010
35.5
At3g02200
38.0
40.5
At1g26630
At1g26630
42.2
At1g70600
At1g23440
43.9
At4g38510
50.6
At1g19330
At1g16790
At1g15690
52.3
At1g15690
At1g15780
54.0
At1g11870
59.0
65.7
At1g09970
At5g60390
At4g01630
At2g20890
At1g05850
At1g04950
73.1
At4g37120
A8
At4g31720a
0.0
u
At1g29580*
p65t90v258
7.1
At1g50240
8.8
At1g35680*
At4g33380
9.7
B/C
At1g31812b
At1g31910
11.4
At4g16510
T
At4g15802
At4g14570b
12.1
At4g17900*b
At4g15510
12.8
At4g16400a
At4g15910b
15.6
At4g04930
17.2
At4g34520*a
U
17.8
e32t66h142
At4g31460
20.3
At4g26840*a
At4g28200
24.5
At4g25140*a
26.8
e48m34h182
29.0
34.3
At1g28560b
B
35.1
At4g38570*
At1g28530b
At1g24310
39.9
p62m39v124
40.5
At1g25540*
At1g14610*
43.8
At1g26850*
44.8
At1g23180a
48.7
At1g19630*
At1g18340a
A
49.4
At1g18060
At1g11880b
56.6
e39t74h760
59.1
At1g11240
60.6
67.8
At1g03180b
69.8
p48t87v295
B/C
T
U
B
A

## Slide 9
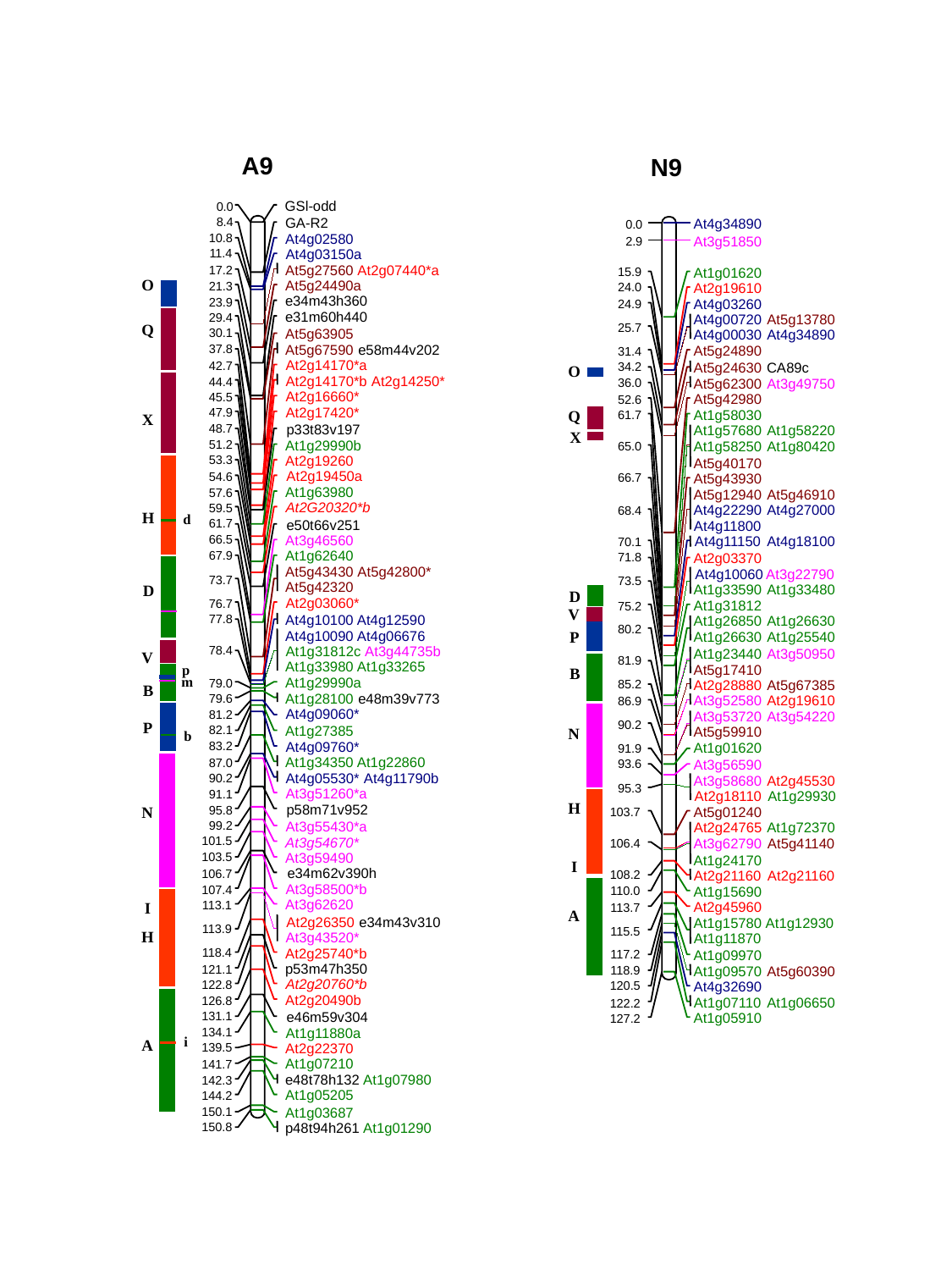

A9
GSl-odd
0.0
8.4
GA-R2
10.8
At4g02580
11.4
At4g03150a
At5g27560
At2g07440*a
17.2
O
Q
X
H
d
D
V
p
m
B
P
b
N
I
H
i
A
At5g24490a
21.3
e34m43h360
23.9
e31m60h440
29.4
30.1
At5g63905
37.8
At5g67590
e58m44v202
At2g14170*a
42.7
At2g14170*b
At2g14250*
44.4
At2g16660*
45.5
At2g17420*
47.9
48.7
p33t83v197
51.2
At1g29990b
53.3
At2g19260
At2g19450a
54.6
At1g63980
57.6
At2G20320*b
59.5
61.7
e50t66v251
66.5
At3g46560
67.9
At1g62640
At5g43430
At5g42800*
73.7
At5g42320
At2g03060*
76.7
77.8
At4g10100
At4g12590
At4g10090
At4g06676
78.4
At1g31812c
At3g44735b
At1g33980
At1g33265
At1g29990a
79.0
At1g28100
e48m39v773
79.6
At4g09060*
81.2
82.1
At1g27385
83.2
At4g09760*
At1g34350
At1g22860
87.0
At4g05530*
At4g11790b
90.2
At3g51260*a
91.1
p58m71v952
95.8
99.2
At3g55430*a
101.5
At3g54670*
103.5
At3g59490
e34m62v390h
106.7
At3g58500*b
107.4
At3g62620
113.1
At2g26350
e34m43v310
113.9
At3g43520*
118.4
At2g25740*b
p53m47h350
121.1
At2g20760*b
122.8
At2g20490b
126.8
131.1
e46m59v304
134.1
At1g11880a
139.5
At2g22370
At1g07210
141.7
e48t78h132
At1g07980
142.3
At1g05205
144.2
150.1
At1g03687
150.8
p48t94h261
At1g01290
N9
At4g34890
0.0
At3g51850
2.9
15.9
At1g01620
24.0
At2g19610
At4g03260
24.9
At4g00720
At5g13780
25.7
At4g00030
At4g34890
At5g24890
31.4
34.2
At5g24630
CA89c
36.0
At5g62300
At3g49750
At5g42980
52.6
At1g58030
61.7
At1g57680
At1g58220
At1g58250
At1g80420
65.0
At5g40170
66.7
At5g43930
At5g12940
At5g46910
At4g22290
At4g27000
68.4
At4g11800
At4g11150
At4g18100
70.1
71.8
At2g03370
At4g10060
At3g22790
73.5
At1g33590
At1g33480
At1g31812
75.2
At1g26850
At1g26630
80.2
At1g26630
At1g25540
At1g23440
At3g50950
81.9
At5g17410
85.2
At2g28880
At5g67385
At3g52580
At2g19610
86.9
At3g53720
At3g54220
90.2
At5g59910
At1g01620
91.9
93.6
At3g56590
At3g58680
At2g45530
95.3
At2g18110
At1g29930
At5g01240
103.7
At2g24765
At1g72370
At3g62790
At5g41140
106.4
At1g24170
108.2
At2g21160
At2g21160
110.0
At1g15690
At2g45960
113.7
At1g15780
At1g12930
115.5
At1g11870
117.2
At1g09970
118.9
At1g09570
At5g60390
120.5
At4g32690
At1g07110
At1g06650
122.2
At1g05910
127.2
O
Q
X
D
V
P
B
N
H
I
A

## Slide 10
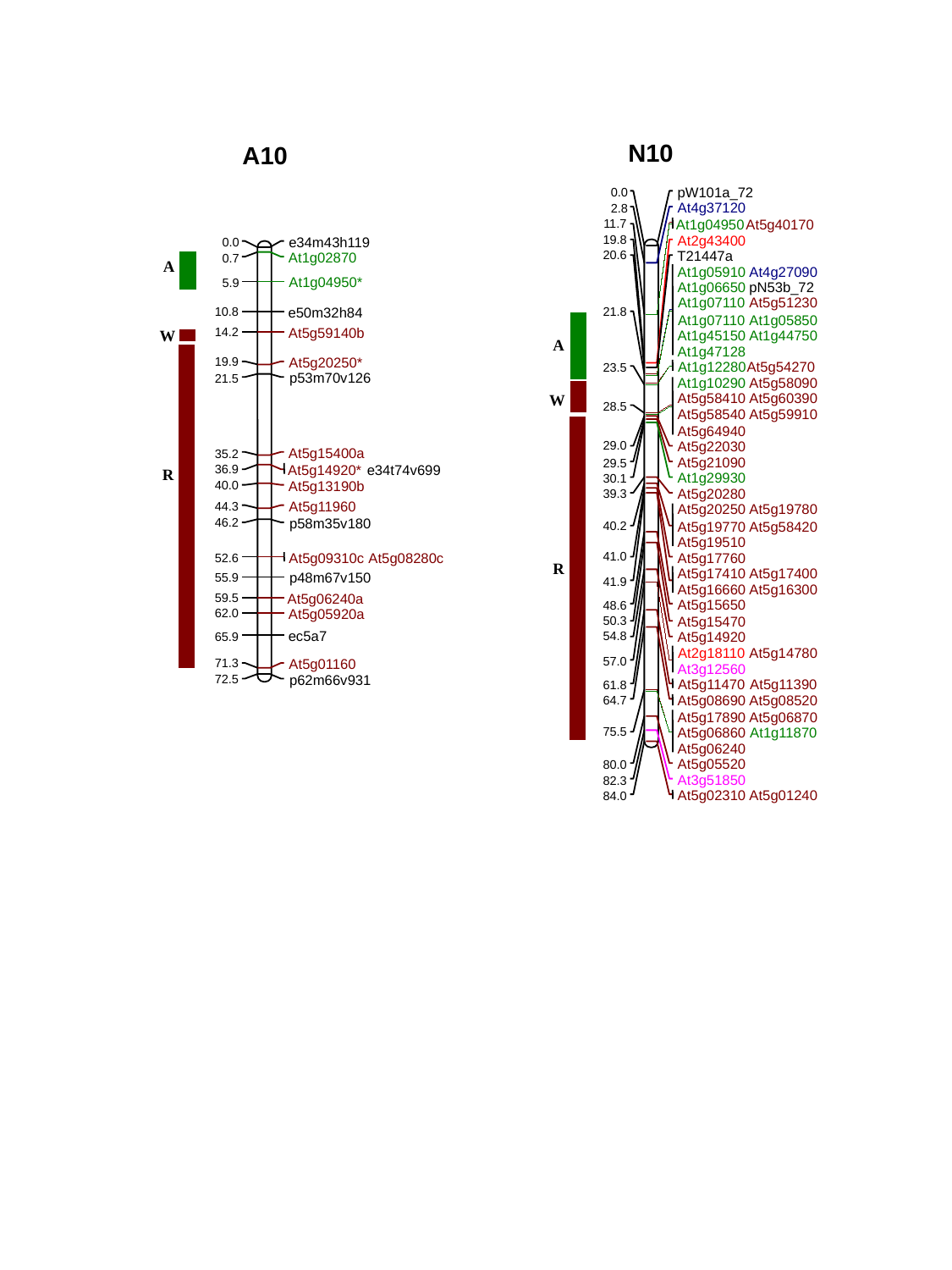

N10
A10
e34m43h119
0.0
At1g02870
0.7
At1g04950*
5.9
10.8
e50m32h84
14.2
At5g59140b
19.9
At5g20250*
p53m70v126
21.5
At5g15400a
35.2
36.9
At5g14920*
e34t74v699
40.0
At5g13190b
At5g11960
44.3
46.2
p58m35v180
At5g09310c
At5g08280c
52.6
55.9
p48m67v150
59.5
At5g06240a
62.0
At5g05920a
ec5a7
65.9
71.3
At5g01160
72.5
p62m66v931
A
W
R
pW101a_72
0.0
At4g37120
2.8
11.7
At1g04950
At5g40170
19.8
At2g43400
20.6
T21447a
At1g05910
At4g27090
At1g06650
pN53b_72
At1g07110
At5g51230
21.8
At1g07110
At1g05850
A
W
R
At1g45150
At1g44750
At1g47128
At1g12280
At5g54270
23.5
At1g10290
At5g58090
At5g58410
At5g60390
28.5
At5g58540
At5g59910
At5g64940
29.0
At5g22030
At5g21090
29.5
At1g29930
30.1
At5g20280
39.3
At5g20250
At5g19780
40.2
At5g19770
At5g58420
At5g19510
41.0
At5g17760
At5g17410
At5g17400
41.9
At5g16660
At5g16300
At5g15650
48.6
50.3
At5g15470
54.8
At5g14920
At2g18110
At5g14780
57.0
At3g12560
At5g11470
At5g11390
61.8
At5g08690
At5g08520
64.7
At5g17890
At5g06870
75.5
At5g06860
At1g11870
At5g06240
At5g05520
80.0
At3g51850
82.3
At5g02310
At5g01240
84.0
