# Supplementary material for: Comparative mapping of Brassica juncea and Arabidopsis thaliana using Intron Polymorphism (IP) markers: homoeologous relationships, diversification and evolution of the A, B and C Brassica genomes
Source: BMC Genomics. 2008 Mar 3;9:113. doi: 10.1186/1471-2164-9-113 (PMC2277410; doi:10.1186/1471-2164-9-113)
Supplement: Additional file 3 — Comparative genome organization of the B genome of B. juncea (B1–B8; present study) and B. nigra (G1–G8) [18]. This file contains the map of the B genome of B. nigra [18] with the RFLP loci converted to their corresponding At (A. thaliana) loci and a detailed comparison (in terms of the At loci arrangement) of this map with the B genome of B. juncea (present study). [file 1471-2164-9-113-S3.ppt]

## Slide 1
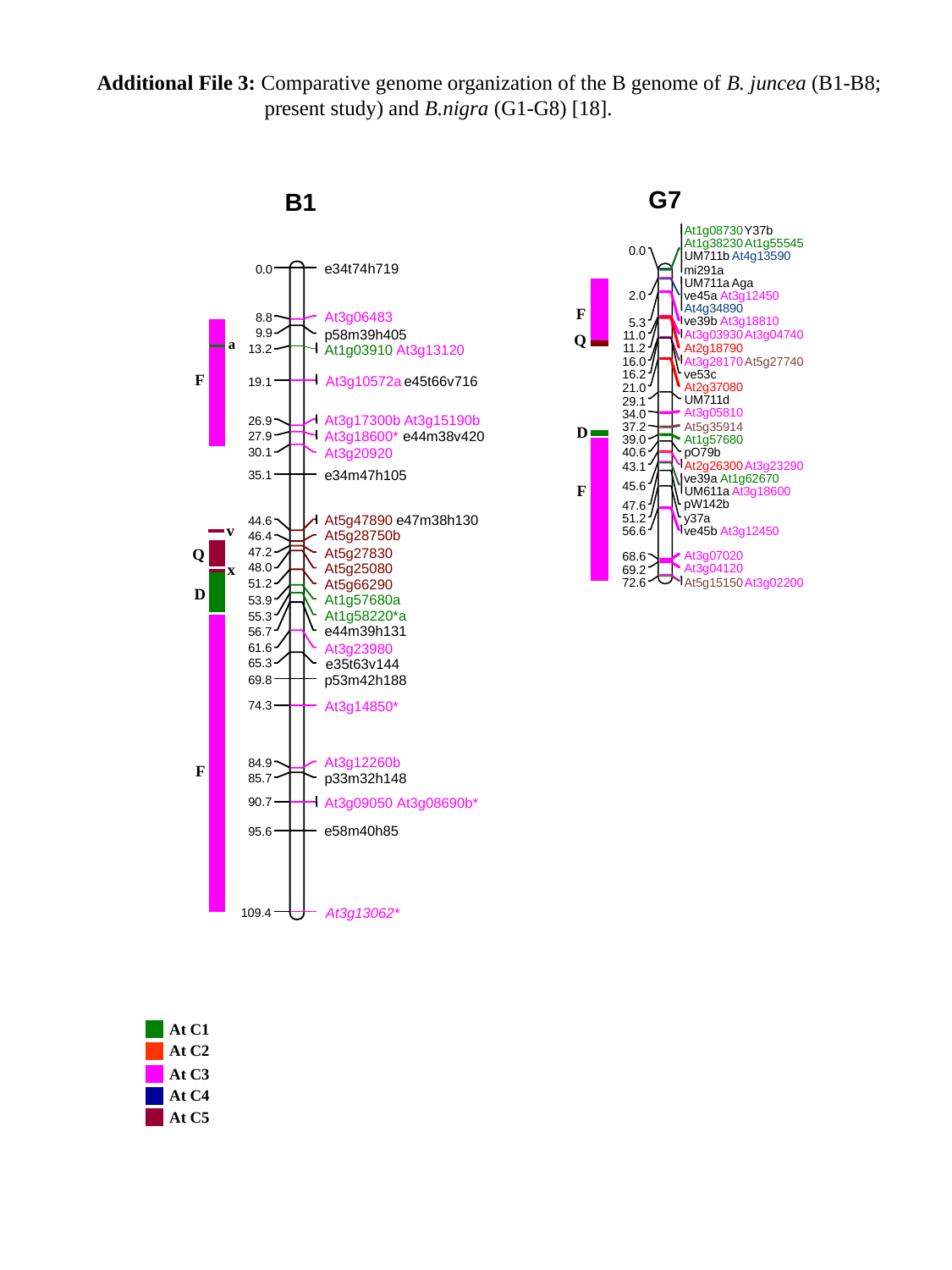

Additional File 3: Comparative genome organization of the B genome of B. juncea (B1-B8; present study) and B.nigra (G1-G8) [18].
G7
At1g08730
Y37b
At1g38230
At1g55545
0.0
UM711b
At4g13590
mi291a
UM711a
Aga
2.0
ve45a
At3g12450
At4g34890
ve39b
At3g18810
5.3
At3g03930
At3g04740
11.0
11.2
At2g18790
16.0
At3g28170
At5g27740
16.2
ve53c
At2g37080
21.0
UM711d
29.1
At3g05810
34.0
37.2
At5g35914
39.0
At1g57680
40.6
pO79b
At2g26300
At3g23290
43.1
ve39a
At1g62670
45.6
UM611a
At3g18600
pW142b
47.6
51.2
y37a
56.6
ve45b
At3g12450
At3g07020
68.6
At3g04120
69.2
72.6
At5g15150
At3g02200
F
Q
D
F
B1
e34t74h719
0.0
At3g06483
8.8
9.9
p58m39h405
13.2
At1g03910
At3g13120
At3g10572a
e45t66v716
19.1
At3g17300b
At3g15190b
26.9
At3g18600*
e44m38v420
27.9
30.1
At3g20920
e34m47h105
35.1
At5g47890
e47m38h130
44.6
At5g28750b
46.4
47.2
At5g27830
48.0
At5g25080
51.2
At5g66290
At1g57680a
53.9
At1g58220*a
55.3
e44m39h131
56.7
61.6
At3g23980
65.3
e35t63v144
p53m42h188
69.8
74.3
At3g14850*
At3g12260b
84.9
p33m32h148
85.7
90.7
At3g09050
At3g08690b*
e58m40h85
95.6
At3g13062*
109.4
a
F
v
Q
x
D
F
At C1
At C2
At C3
At C4
At C5

## Slide 2
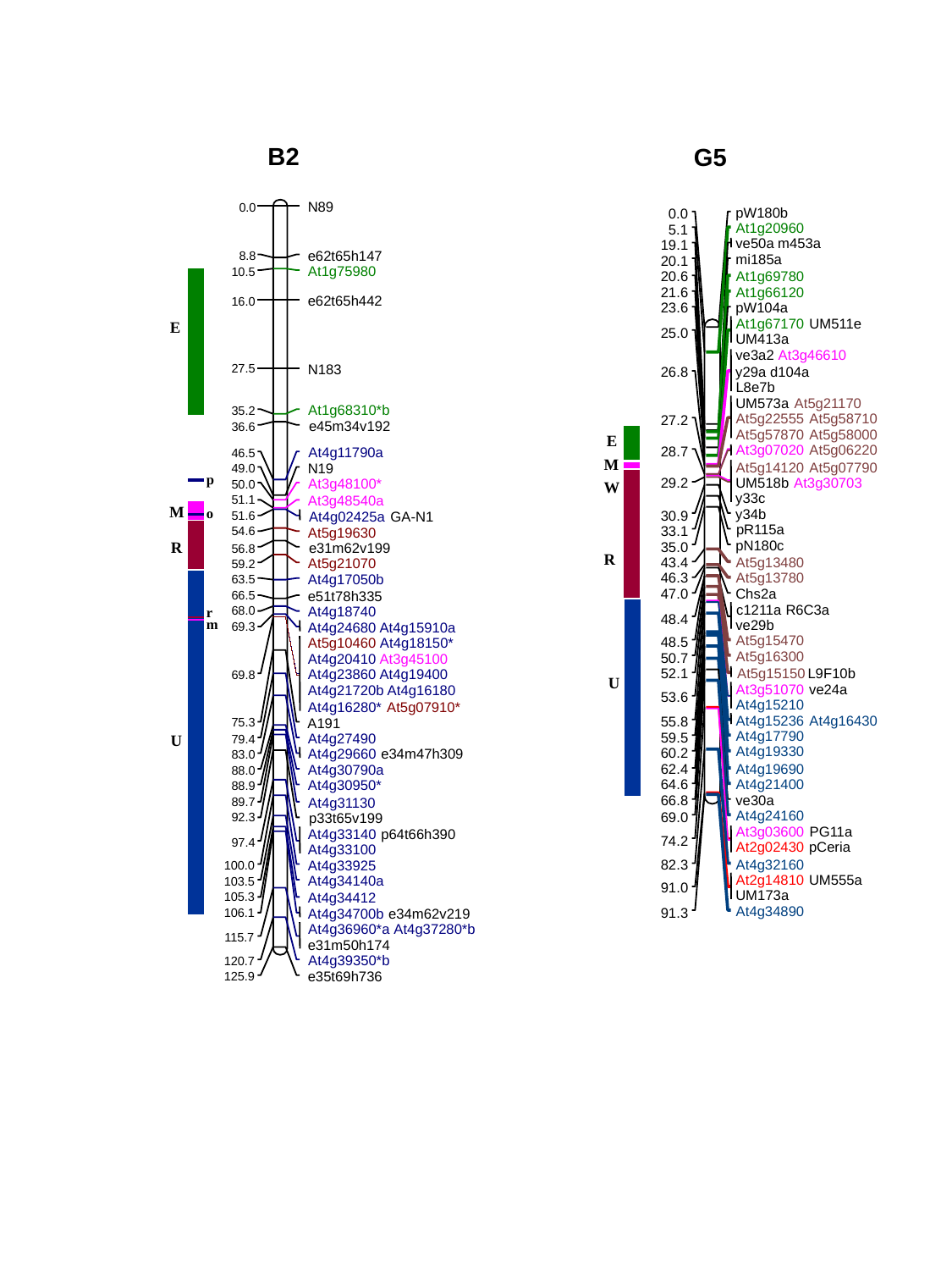

B2
N89
0.0
e62t65h147
8.8
At1g75980
10.5
e62t65h442
16.0
27.5
N183
At1g68310*b
35.2
e45m34v192
36.6
At4g11790a
46.5
N19
49.0
At3g48100*
50.0
51.1
At3g48540a
51.6
At4g02425a
GA-N1
54.6
At5g19630
e31m62v199
56.8
At5g21070
59.2
At4g17050b
63.5
66.5
e51t78h335
68.0
At4g18740
69.3
At4g24680
At4g15910a
At5g10460
At4g18150*
At4g20410
At3g45100
At4g23860
At4g19400
69.8
At4g21720b
At4g16180
At4g16280*
At5g07910*
75.3
A191
At4g27490
79.4
At4g29660
e34m47h309
83.0
At4g30790a
88.0
At4g30950*
88.9
89.7
At4g31130
92.3
p33t65v199
At4g33140
p64t66h390
97.4
At4g33100
At4g33925
100.0
At4g34140a
103.5
105.3
At4g34412
106.1
At4g34700b
e34m62v219
At4g36960*a
At4g37280*b
115.7
e31m50h174
At4g39350*b
120.7
e35t69h736
125.9
E
p
M
o
R
r
m
U
G5
pW180b
0.0
At1g20960
5.1
ve50a
m453a
19.1
mi185a
20.1
20.6
At1g69780
21.6
At1g66120
23.6
pW104a
At1g67170
UM511e
25.0
UM413a
ve3a2
At3g46610
26.8
y29a
d104a
L8e7b
UM573a
At5g21170
At5g22555
At5g58710
27.2
At5g57870
At5g58000
At3g07020
At5g06220
28.7
At5g14120
At5g07790
29.2
UM518b
At3g30703
y33c
y34b
30.9
pR115a
33.1
pN180c
35.0
43.4
At5g13480
46.3
At5g13780
47.0
Chs2a
c1211a
R6C3a
48.4
ve29b
At5g15470
48.5
At5g16300
50.7
52.1
At5g15150
L9F10b
At3g51070
ve24a
53.6
At4g15210
At4g15236
At4g16430
55.8
At4g17790
59.5
At4g19330
60.2
62.4
At4g19690
64.6
At4g21400
66.8
ve30a
At4g24160
69.0
At3g03600
PG11a
74.2
At2g02430
pCeria
82.3
At4g32160
At2g14810
UM555a
91.0
UM173a
At4g34890
91.3
E
M
W
R
U

## Slide 3
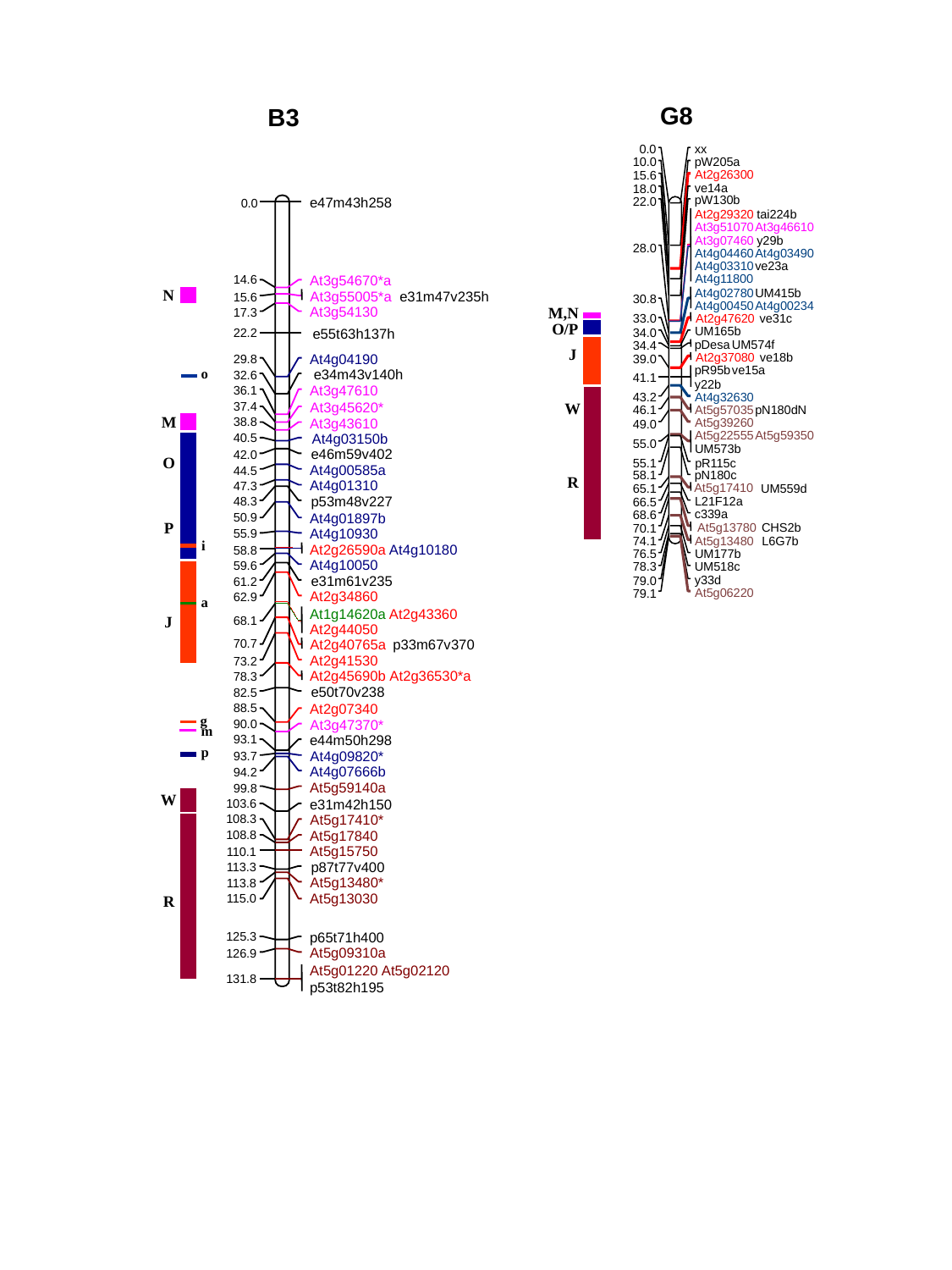

G8
B3
e47m43h258
0.0
14.6
At3g54670*a
At3g55005*a
e31m47v235h
15.6
At3g54130
17.3
22.2
e55t63h137h
At4g04190
29.8
e34m43v140h
32.6
At3g47610
36.1
37.4
At3g45620*
38.8
At3g43610
40.5
 At4g03150b
e46m59v402
42.0
At4g00585a
44.5
At4g01310
47.3
p53m48v227
48.3
50.9
At4g01897b
55.9
At4g10930
At2g26590a
At4g10180
58.8
At4g10050
59.6
e31m61v235
61.2
At2g34860
62.9
At1g14620a
At2g43360
68.1
At2g44050
70.7
At2g40765a
p33m67v370
At2g41530
73.2
At2g45690b
At2g36530*a
78.3
e50t70v238
82.5
88.5
At2g07340
90.0
At3g47370*
93.1
e44m50h298
At4g09820*
93.7
At4g07666b
94.2
At5g59140a
99.8
103.6
e31m42h150
108.3
At5g17410*
108.8
At5g17840
At5g15750
110.1
p87t77v400
113.3
At5g13480*
113.8
At5g13030
115.0
125.3
p65t71h400
At5g09310a
126.9
At5g01220
At5g02120
131.8
p53t82h195
N
o
M
O
P
i
a
J
g
m
p
W
R
0.0
xx
10.0
pW205a
At2g26300
15.6
ve14a
18.0
pW130b
22.0
At2g29320
tai224b
At3g51070
At3g46610
At3g07460
y29b
28.0
At4g04460
At4g03490
At4g03310
ve23a
At4g11800
At4g02780
UM415b
30.8
At4g00450
At4g00234
33.0
At2g47620
ve31c
UM165b
34.0
pDesa
UM574f
34.4
At2g37080
ve18b
39.0
pR95b
ve15a
41.1
y22b
43.2
At4g32630
46.1
At5g57035
pN180dN
At5g39260
49.0
At5g22555
At5g59350
55.0
UM573b
55.1
pR115c
58.1
pN180c
At5g17410
65.1
UM559d
L21F12a
66.5
c339a
68.6
At5g13780
CHS2b
70.1
74.1
At5g13480
L6G7b
76.5
UM177b
78.3
UM518c
y33d
79.0
At5g06220
79.1
M,N
O/P
J
W
R

## Slide 4
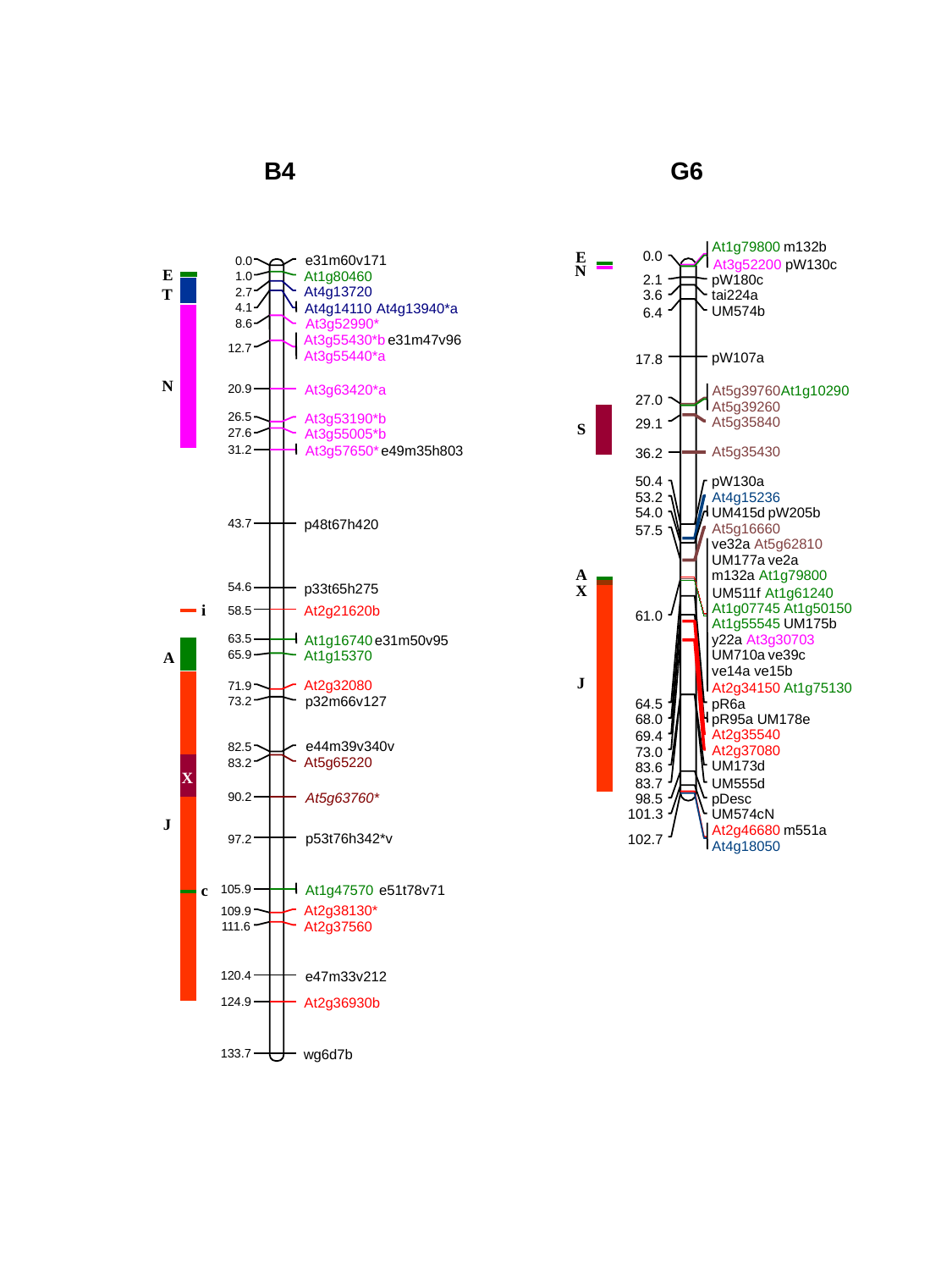

B4
e31m60v171
0.0
E
T
N
i
A
X
J
c
At1g80460
1.0
At4g13720
2.7
4.1
At4g14110
At4g13940*a
8.6
 At3g52990*
At3g55430*b
e31m47v96
12.7
At3g55440*a
20.9
At3g63420*a
26.5
At3g53190*b
27.6
At3g55005*b
31.2
At3g57650*
e49m35h803
43.7
p48t67h420
54.6
p33t65h275
At2g21620b
58.5
63.5
At1g16740
e31m50v95
65.9
At1g15370
At2g32080
71.9
p32m66v127
73.2
e44m39v340v
82.5
At5g65220
83.2
90.2
 At5g63760*
p53t76h342*v
97.2
105.9
At1g47570
e51t78v71
At2g38130*
109.9
At2g37560
111.6
120.4
e47m33v212
124.9
At2g36930b
133.7
wg6d7b
G6
At1g79800
m132b
0.0
At3g52200
pW130c
2.1
pW180c
3.6
tai224a
UM574b
6.4
pW107a
17.8
At5g39760
At1g10290
27.0
At5g39260
At5g35840
29.1
At5g35430
36.2
50.4
pW130a
53.2
At4g15236
54.0
UM415d
pW205b
At5g16660
57.5
ve32a
At5g62810
UM177a
ve2a
m132a
At1g79800
UM511f
At1g61240
At1g07745
At1g50150
61.0
At1g55545
UM175b
y22a
At3g30703
UM710a
ve39c
ve14a
ve15b
At2g34150
At1g75130
64.5
pR6a
68.0
pR95a
UM178e
At2g35540
69.4
At2g37080
73.0
UM173d
83.6
83.7
UM555d
98.5
pDesc
UM574cN
At2g46680
m551a
At4g18050
E
N
S
X
J
101.3
102.7
A

## Slide 5
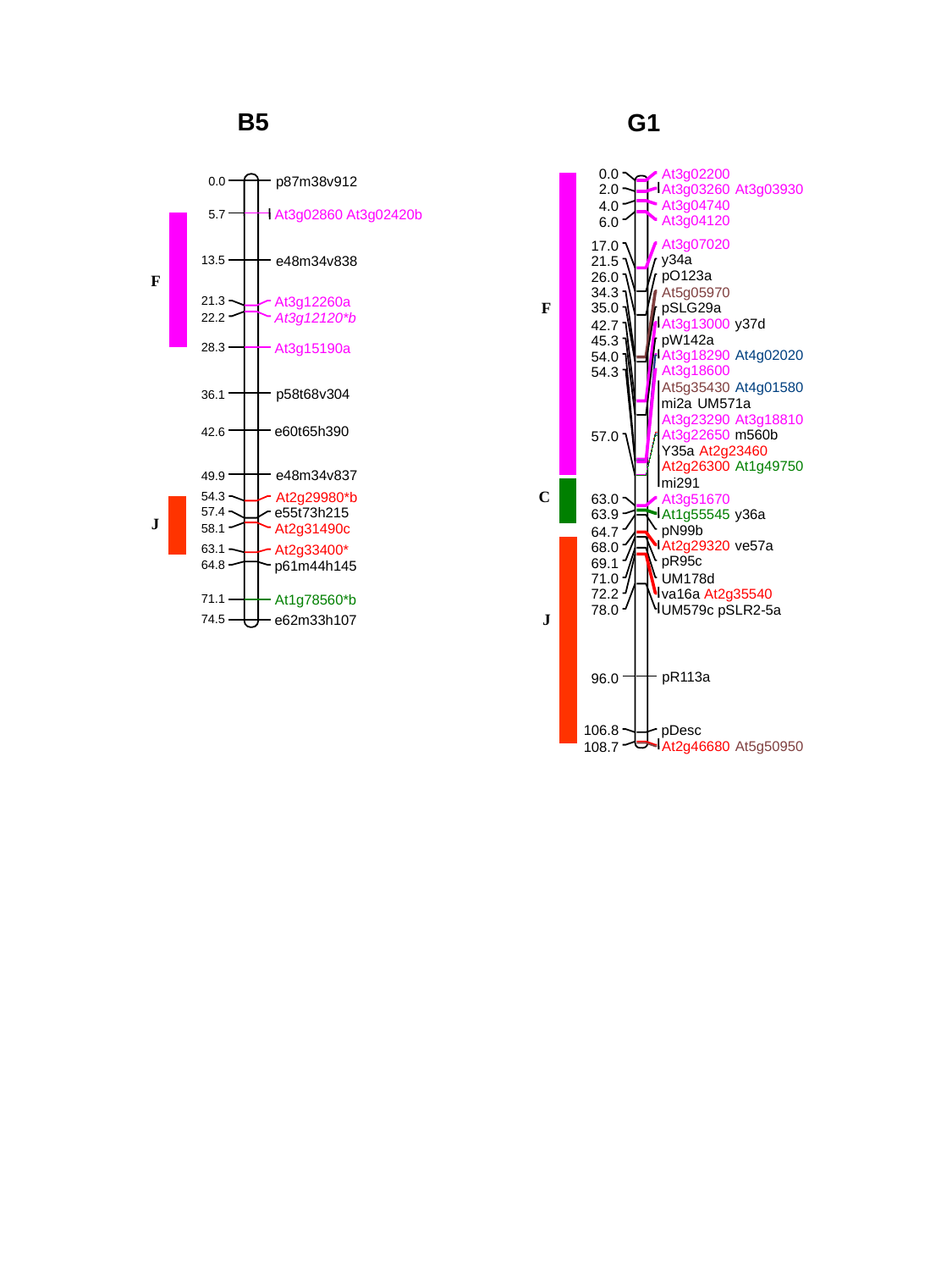

B5
p87m38v912
0.0
At3g02860
At3g02420b
5.7
13.5
e48m34v838
F
21.3
At3g12260a
At3g12120*b
22.2
28.3
At3g15190a
p58t68v304
36.1
e60t65h390
42.6
e48m34v837
49.9
54.3
At2g29980*b
57.4
e55t73h215
J
At2g31490c
58.1
63.1
At2g33400*
64.8
p61m44h145
71.1
At1g78560*b
74.5
e62m33h107
G1
0.0
At3g02200
2.0
At3g03260
At3g03930
At3g04740
4.0
At3g04120
6.0
At3g07020
17.0
y34a
21.5
pO123a
26.0
34.3
At5g05970
35.0
pSLG29a
At3g13000
y37d
42.7
pW142a
45.3
At3g18290
At4g02020
54.0
At3g18600
54.3
At5g35430
At4g01580
mi2a
UM571a
At3g23290
At3g18810
At3g22650
m560b
57.0
Y35a
At2g23460
At2g26300
At1g49750
mi291
63.0
At3g51670
63.9
At1g55545
y36a
pN99b
64.7
At2g29320
ve57a
68.0
pR95c
69.1
71.0
UM178d
72.2
va16a
At2g35540
78.0
UM579c
pSLR2-5a
pR113a
96.0
106.8
pDesc
At2g46680
At5g50950
108.7
F
C
J

## Slide 6
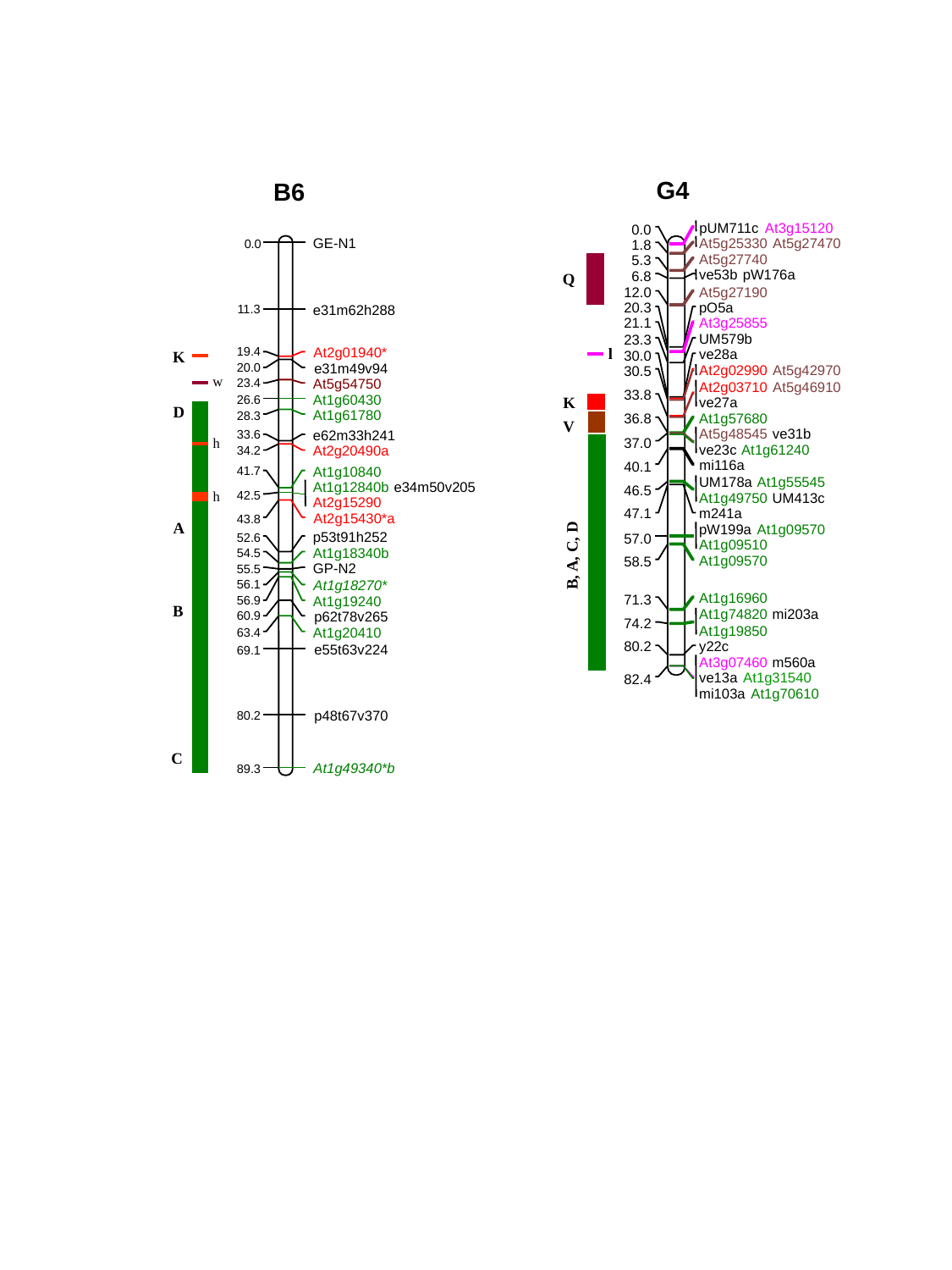

G4
pUM711c
At3g15120
0.0
At5g25330
At5g27470
1.8
At5g27740
5.3
ve53b
pW176a
6.8
12.0
At5g27190
20.3
pO5a
21.1
At3g25855
UM579b
23.3
ve28a
30.0
At2g02990
At5g42970
30.5
At2g03710
At5g46910
33.8
ve27a
36.8
At1g57680
At5g48545
ve31b
37.0
ve23c
At1g61240
mi116a
40.1
UM178a
At1g55545
46.5
At1g49750
UM413c
47.1
m241a
pW199a
At1g09570
57.0
At1g09510
At1g09570
58.5
At1g16960
71.3
At1g74820
mi203a
74.2
At1g19850
80.2
y22c
At3g07460
m560a
ve13a
At1g31540
82.4
mi103a
At1g70610
B6
GE-N1
0.0
11.3
e31m62h288
K
19.4
At2g01940*
20.0
e31m49v94
w
23.4
At5g54750
At1g60430
26.6
D
At1g61780
28.3
33.6
e62m33h241
h
34.2
At2g20490a
41.7
At1g10840
At1g12840b
e34m50v205
h
42.5
At2g15290
At2g15430*a
A
43.8
p53t91h252
52.6
At1g18340b
54.5
GP-N2
55.5
56.1
At1g18270*
56.9
At1g19240
B
60.9
p62t78v265
At1g20410
63.4
e55t63v224
69.1
p48t67v370
80.2
C
At1g49340*b
89.3
Q
l
K
V
B, A, C, D

## Slide 7
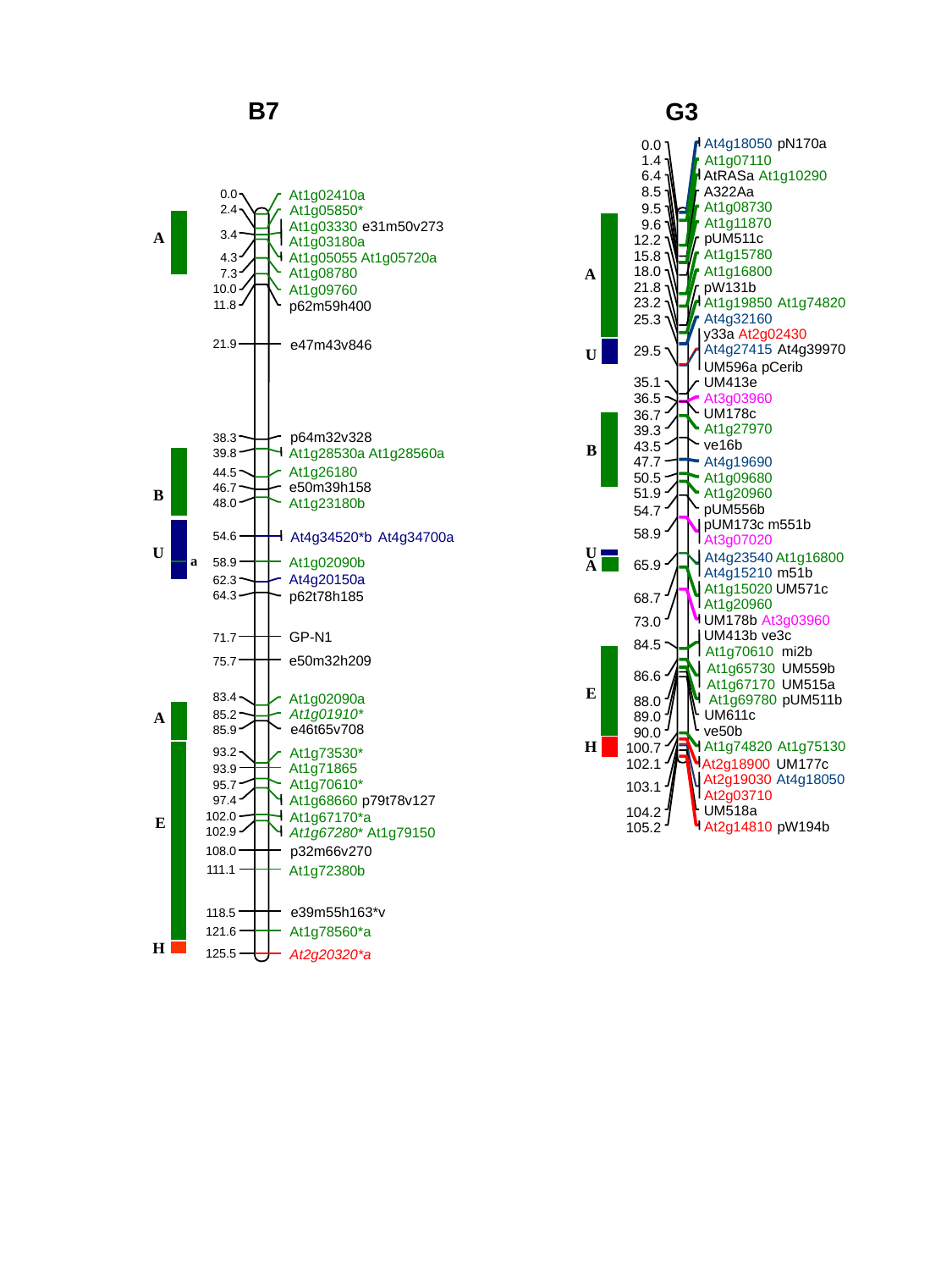

B7
0.0
At1g02410a
2.4
At1g05850*
At1g03330
e31m50v273
3.4
At1g03180a
At1g05055
At1g05720a
4.3
At1g08780
7.3
10.0
At1g09760
11.8
p62m59h400
21.9
e47m43v846
p64m32v328
38.3
At1g28530a
At1g28560a
39.8
At1g26180
44.5
e50m39h158
46.7
At1g23180b
48.0
54.6
At4g34520*b
At4g34700a
At1g02090b
58.9
At4g20150a
62.3
64.3
p62t78h185
GP-N1
71.7
e50m32h209
75.7
83.4
At1g02090a
At1g01910*
85.2
e46t65v708
85.9
93.2
At1g73530*
At1g71865
93.9
At1g70610*
95.7
At1g68660
p79t78v127
97.4
102.0
At1g67170*a
102.9
At1g67280*
At1g79150
108.0
p32m66v270
111.1
At1g72380b
e39m55h163*v
118.5
At1g78560*a
121.6
125.5
At2g20320*a
A
B
U
a
A
E
H
G3
At4g18050
pN170a
0.0
1.4
At1g07110
6.4
AtRASa
At1g10290
8.5
A322Aa
At1g08730
9.5
At1g11870
9.6
pUM511c
12.2
At1g15780
15.8
18.0
At1g16800
21.8
pW131b
23.2
At1g19850
At1g74820
At4g32160
25.3
y33a
At2g02430
At4g27415
At4g39970
29.5
UM596a
pCerib
35.1
UM413e
36.5
At3g03960
UM178c
36.7
At1g27970
39.3
ve16b
43.5
47.7
At4g19690
50.5
At1g09680
51.9
At1g20960
pUM556b
54.7
pUM173c
m551b
58.9
At3g07020
At4g23540
At1g16800
65.9
At4g15210
m51b
At1g15020
UM571c
68.7
At1g20960
UM178b
At3g03960
73.0
UM413b
ve3c
84.5
At1g70610
mi2b
At1g65730
UM559b
86.6
At1g67170
UM515a
At1g69780
pUM511b
88.0
UM611c
89.0
ve50b
90.0
At1g74820
At1g75130
100.7
102.1
At2g18900
UM177c
At2g19030
At4g18050
103.1
At2g03710
UM518a
104.2
At2g14810
pW194b
105.2
A
U
B
U
A
E
H

## Slide 8
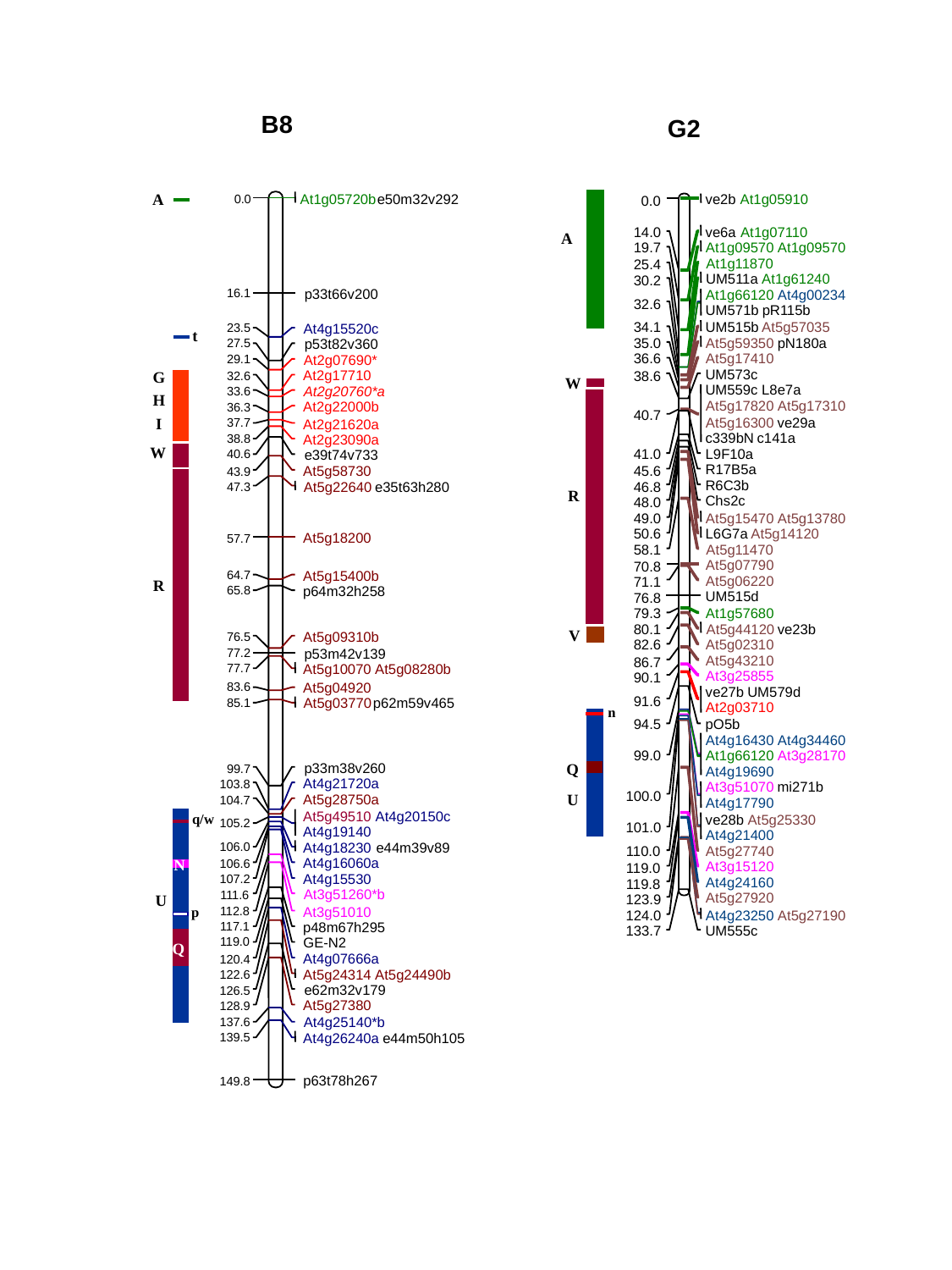

B8
At1g05720b
e50m32v292
0.0
16.1
p33t66v200
23.5
At4g15520c
27.5
p53t82v360
29.1
At2g07690*
At2g17710
32.6
At2g20760*a
33.6
At2g22000b
36.3
37.7
At2g21620a
38.8
At2g23090a
40.6
e39t74v733
At5g58730
43.9
At5g22640
e35t63h280
47.3
At5g18200
57.7
64.7
At5g15400b
65.8
p64m32h258
At5g09310b
76.5
77.2
p53m42v139
77.7
At5g10070
At5g08280b
83.6
At5g04920
At5g03770
p62m59v465
85.1
p33m38v260
99.7
At4g21720a
103.8
At5g28750a
104.7
At5g49510
At4g20150c
105.2
At4g19140
106.0
At4g18230
e44m39v89
At4g16060a
106.6
At4g15530
107.2
At3g51260*b
111.6
112.8
At3g51010
117.1
p48m67h295
119.0
GE-N2
At4g07666a
120.4
At5g24314
At5g24490b
122.6
e62m32v179
126.5
At5g27380
128.9
137.6
At4g25140*b
139.5
At4g26240a
e44m50h105
p63t78h267
149.8
A
t
G
H
I
W
R
q/w
N
U
p
Q
G2
ve2b
At1g05910
0.0
14.0
ve6a
At1g07110
19.7
At1g09570
At1g09570
At1g11870
25.4
UM511a
At1g61240
30.2
At1g66120
At4g00234
32.6
UM571b
pR115b
34.1
UM515b
At5g57035
35.0
At5g59350
pN180a
36.6
At5g17410
UM573c
38.6
UM559c
L8e7a
At5g17820
At5g17310
40.7
At5g16300
ve29a
c339bN
c141a
41.0
L9F10a
R17B5a
45.6
R6C3b
46.8
Chs2c
48.0
49.0
At5g15470
At5g13780
50.6
L6G7a
At5g14120
58.1
At5g11470
At5g07790
70.8
At5g06220
71.1
UM515d
76.8
79.3
At1g57680
80.1
At5g44120
ve23b
82.6
At5g02310
At5g43210
86.7
At3g25855
90.1
ve27b
UM579d
91.6
At2g03710
94.5
pO5b
At4g16430
At4g34460
99.0
At1g66120
At3g28170
At4g19690
At3g51070
mi271b
100.0
At4g17790
ve28b
At5g25330
101.0
At4g21400
110.0
At5g27740
At3g15120
119.0
At4g24160
119.8
At5g27920
123.9
124.0
At4g23250
At5g27190
133.7
UM555c
A
W
R
V
Q
U
n
