# Supplementary material for: Comparative mapping of Brassica juncea and Arabidopsis thaliana using Intron Polymorphism (IP) markers: homoeologous relationships, diversification and evolution of the A, B and C Brassica genomes
Source: BMC Genomics. 2008 Mar 3;9:113. doi: 10.1186/1471-2164-9-113 (PMC2277410; doi:10.1186/1471-2164-9-113)
Supplement: Additional file 4 — The C genome map of B. napus [16] with the RFLP probes converted to their corresponding At loci. [file 1471-2164-9-113-S4.ppt]

## Slide 1
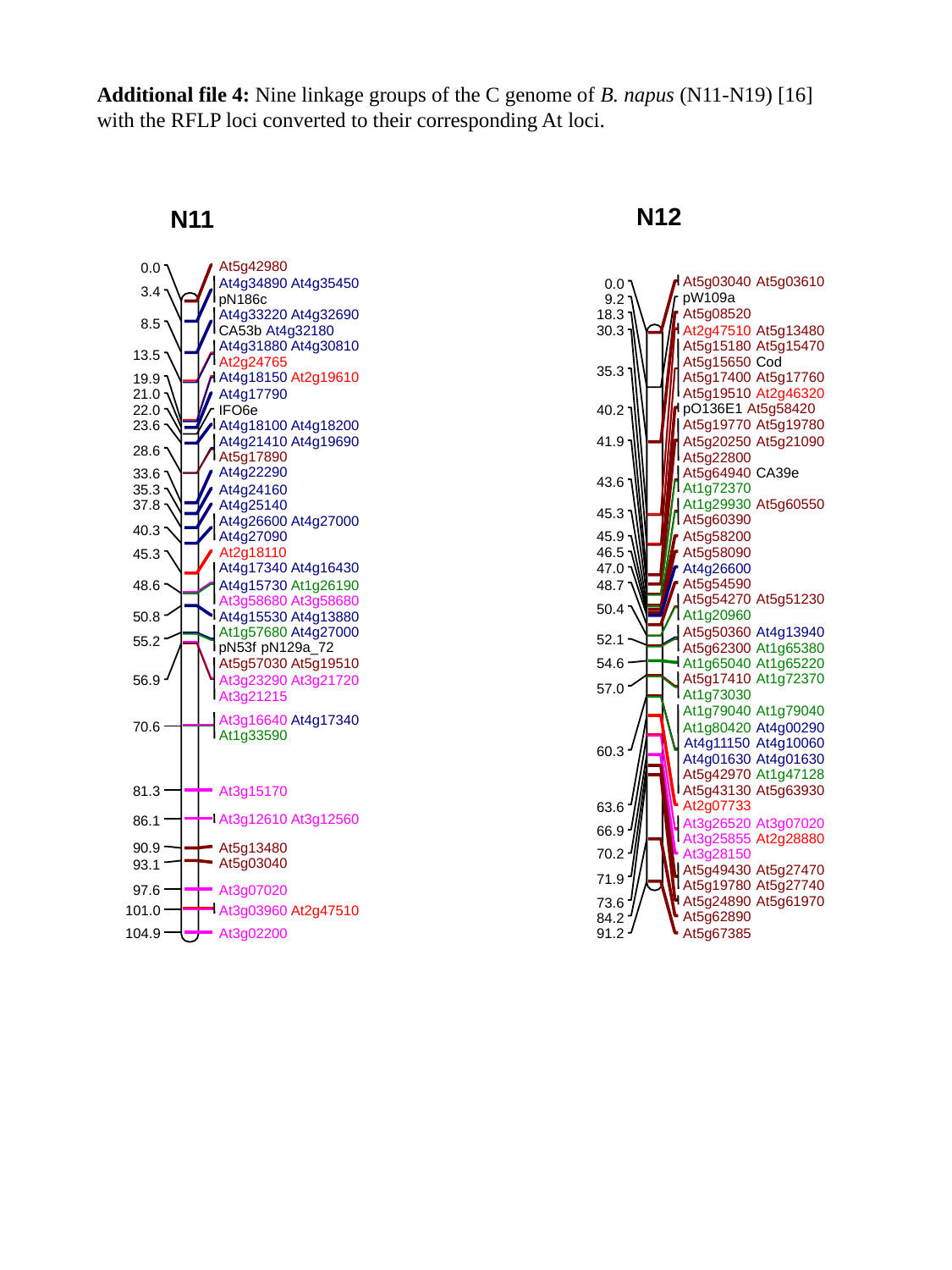

Additional file 4: Nine linkage groups of the C genome of B. napus (N11-N19) [16] with the RFLP loci converted to their corresponding At loci.
N12
N11
At5g42980
0.0
At4g34890
At4g35450
3.4
pN186c
At4g33220
At4g32690
8.5
CA53b
At4g32180
At4g31880
At4g30810
13.5
At2g24765
At4g18150
At2g19610
19.9
21.0
At4g17790
22.0
IFO6e
23.6
At4g18100
At4g18200
At4g21410
At4g19690
28.6
At5g17890
At4g22290
33.6
35.3
At4g24160
37.8
At4g25140
At4g26600
At4g27000
40.3
At4g27090
At2g18110
45.3
At4g17340
At4g16430
48.6
At4g15730
At1g26190
At3g58680
At3g58680
50.8
At4g15530
At4g13880
At1g57680
At4g27000
55.2
pN53f
pN129a_72
At5g57030
At5g19510
56.9
At3g23290
At3g21720
At3g21215
At3g16640
At4g17340
70.6
At1g33590
81.3
At3g15170
At3g12610
At3g12560
86.1
90.9
At5g13480
At5g03040
93.1
97.6
At3g07020
101.0
At3g03960
At2g47510
104.9
At3g02200
At5g03040
At5g03610
0.0
pW109a
9.2
At5g08520
18.3
30.3
At2g47510
At5g13480
At5g15180
At5g15470
At5g15650
Cod
35.3
At5g17400
At5g17760
At5g19510
At2g46320
pO136E1
At5g58420
40.2
At5g19770
At5g19780
41.9
At5g20250
At5g21090
At5g22800
At5g64940
CA39e
43.6
At1g72370
At1g29930
At5g60550
45.3
At5g60390
45.9
At5g58200
46.5
At5g58090
47.0
At4g26600
At5g54590
48.7
At5g54270
At5g51230
50.4
At1g20960
At5g50360
At4g13940
52.1
At5g62300
At1g65380
54.6
At1g65040
At1g65220
At5g17410
At1g72370
57.0
At1g73030
At1g79040
At1g79040
At1g80420
At4g00290
At4g11150
At4g10060
60.3
At4g01630
At4g01630
At5g42970
At1g47128
At5g43130
At5g63930
At2g07733
63.6
At3g26520
At3g07020
66.9
At3g25855
At2g28880
70.2
At3g28150
At5g49430
At5g27470
71.9
At5g19780
At5g27740
At5g24890
At5g61970
73.6
At5g62890
84.2
91.2
At5g67385

## Slide 2
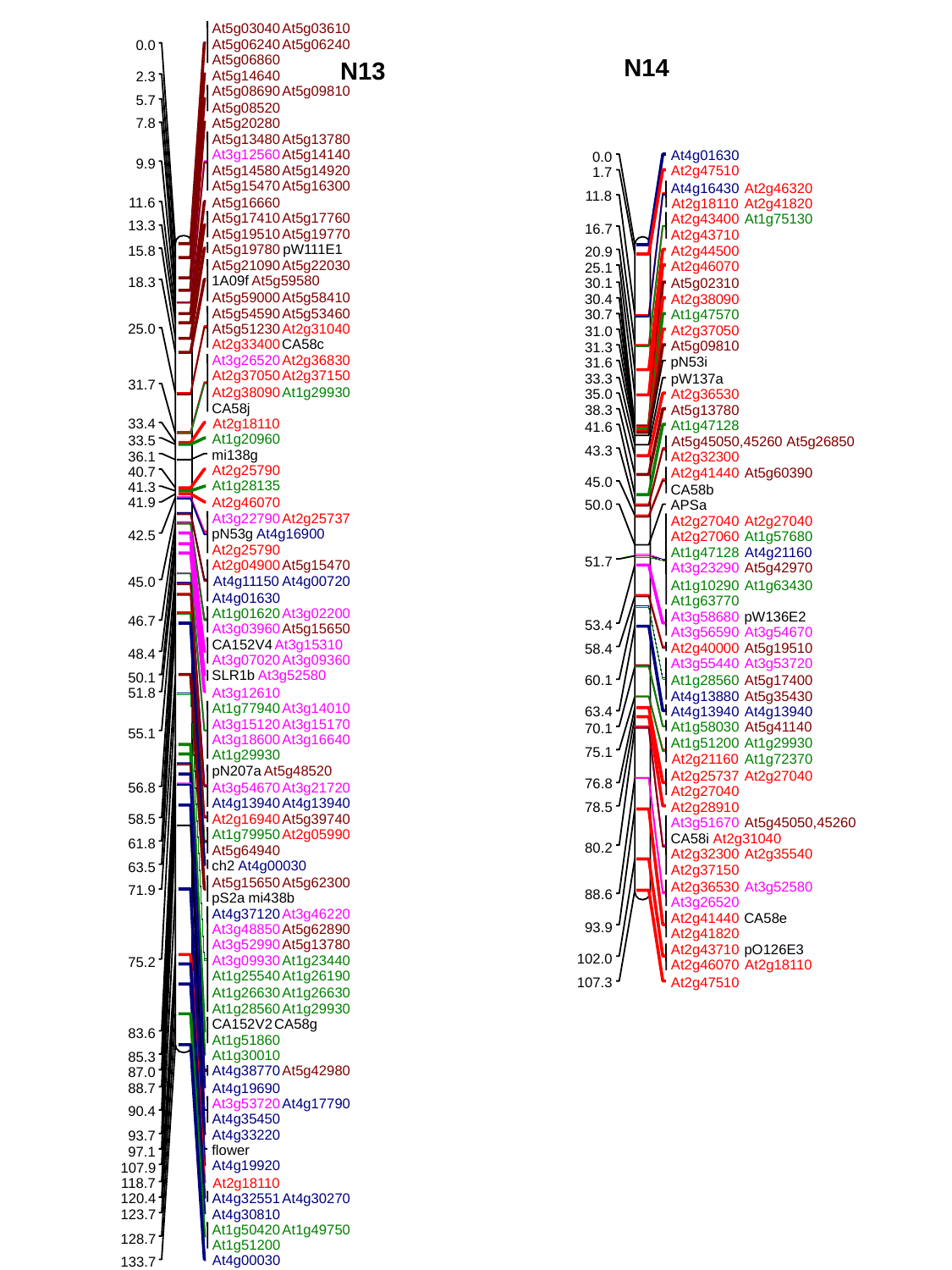

At5g03040
At5g03610
At5g06240
At5g06240
0.0
At5g06860
At5g14640
2.3
At5g08690
At5g09810
5.7
At5g08520
7.8
At5g20280
At5g13480
At5g13780
At3g12560
At5g14140
9.9
At5g14580
At5g14920
At5g15470
At5g16300
11.6
At5g16660
At5g17410
At5g17760
13.3
At5g19510
At5g19770
At5g19780
pW111E1
15.8
At5g21090
At5g22030
1A09f
At5g59580
18.3
At5g59000
At5g58410
At5g54590
At5g53460
25.0
At5g51230
At2g31040
At2g33400
CA58c
At3g26520
At2g36830
At2g37050
At2g37150
31.7
At2g38090
At1g29930
CA58j
33.4
At2g18110
At1g20960
33.5
mi138g
36.1
At2g25790
40.7
At1g28135
41.3
41.9
At2g46070
At3g22790
At2g25737
pN53g
At4g16900
42.5
At2g25790
At2g04900
At5g15470
At4g11150
At4g00720
45.0
At4g01630
At1g01620
At3g02200
46.7
At3g03960
At5g15650
CA152V4
At3g15310
48.4
At3g07020
At3g09360
SLR1b
At3g52580
50.1
51.8
At3g12610
At1g77940
At3g14010
At3g15120
At3g15170
55.1
At3g18600
At3g16640
At1g29930
pN207a
At5g48520
56.8
At3g54670
At3g21720
At4g13940
At4g13940
58.5
At2g16940
At5g39740
At1g79950
At2g05990
61.8
At5g64940
ch2
At4g00030
63.5
At5g15650
At5g62300
71.9
pS2a
mi438b
At4g37120
At3g46220
At3g48850
At5g62890
At3g52990
At5g13780
At3g09930
At1g23440
75.2
At1g25540
At1g26190
At1g26630
At1g26630
At1g28560
At1g29930
CA152V2
CA58g
83.6
At1g51860
At1g30010
85.3
At4g38770
At5g42980
87.0
88.7
At4g19690
At3g53720
At4g17790
90.4
At4g35450
At4g33220
93.7
flower
97.1
At4g19920
107.9
118.7
At2g18110
120.4
At4g32551
At4g30270
123.7
At4g30810
At1g50420
At1g49750
128.7
At1g51200
At4g00030
133.7
N14
N13
At4g01630
0.0
At2g47510
1.7
At4g16430
At2g46320
11.8
At2g18110
At2g41820
At2g43400
At1g75130
16.7
At2g43710
At2g44500
20.9
At2g46070
25.1
30.1
At5g02310
30.4
At2g38090
30.7
At1g47570
At2g37050
31.0
At5g09810
31.3
pN53i
31.6
33.3
pW137a
35.0
At2g36530
38.3
At5g13780
At1g47128
41.6
At5g45050,45260
At5g26850
43.3
At2g32300
At2g41440
At5g60390
45.0
CA58b
50.0
APSa
At2g27040
At2g27040
At2g27060
At1g57680
At1g47128
At4g21160
51.7
At3g23290
At5g42970
At1g10290
At1g63430
At1g63770
At3g58680
pW136E2
53.4
At3g56590
At3g54670
At2g40000
At5g19510
58.4
At3g55440
At3g53720
60.1
At1g28560
At5g17400
At4g13880
At5g35430
63.4
At4g13940
At4g13940
At1g58030
At5g41140
70.1
At1g51200
At1g29930
75.1
At2g21160
At1g72370
At2g25737
At2g27040
76.8
At2g27040
78.5
At2g28910
At3g51670
At5g45050,45260
CA58i
At2g31040
80.2
At2g32300
At2g35540
At2g37150
At2g36530
At3g52580
88.6
At3g26520
At2g41440
CA58e
93.9
At2g41820
At2g43710
pO126E3
102.0
At2g46070
At2g18110
107.3
At2g47510

## Slide 3
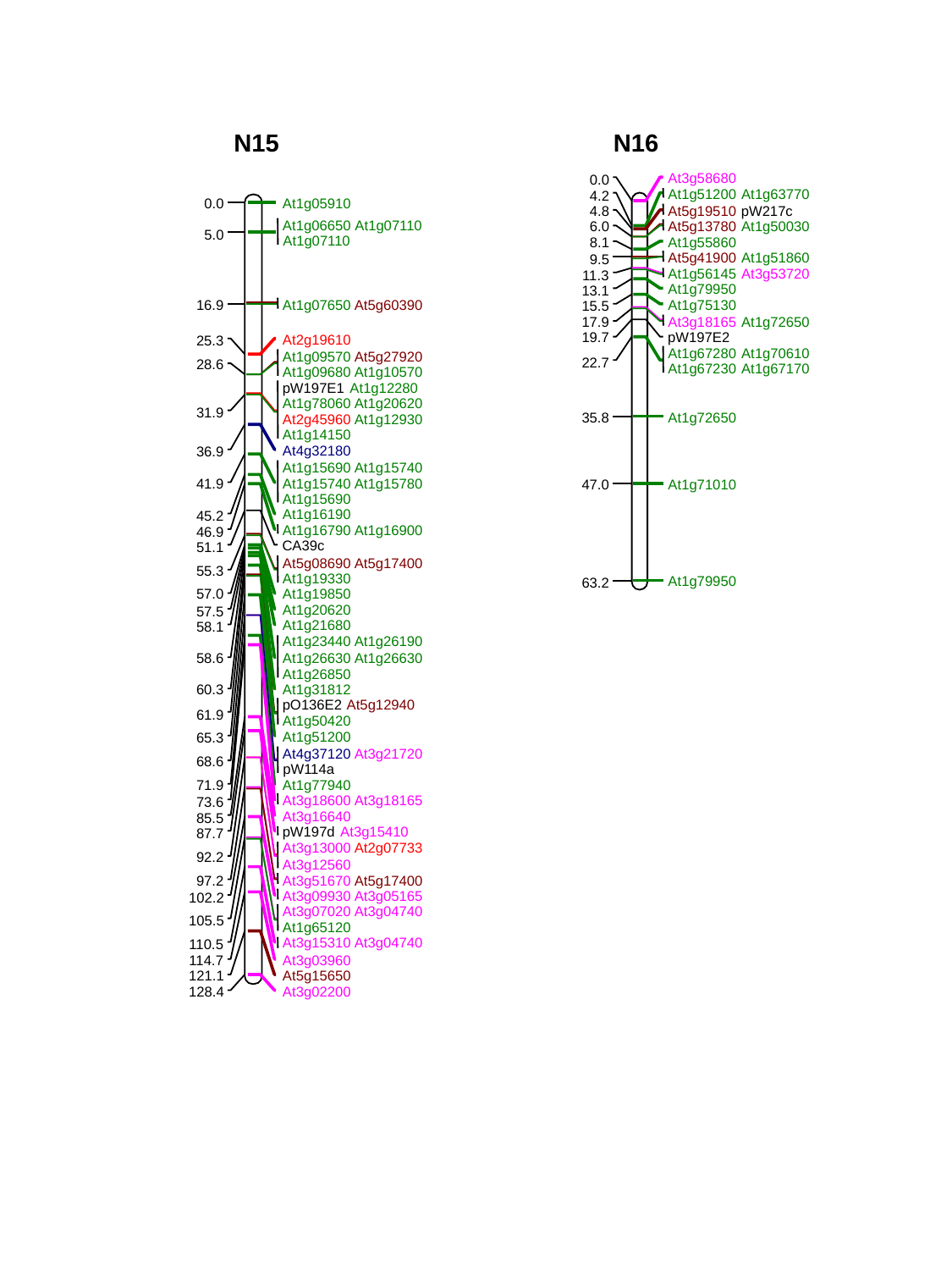

N15
0.0
At1g05910
At1g06650
At1g07110
5.0
At1g07110
16.9
At1g07650
At5g60390
At2g19610
25.3
At1g09570
At5g27920
28.6
At1g09680
At1g10570
pW197E1
At1g12280
At1g78060
At1g20620
31.9
At2g45960
At1g12930
At1g14150
At4g32180
36.9
At1g15690
At1g15740
41.9
At1g15740
At1g15780
At1g15690
At1g16190
45.2
At1g16790
At1g16900
46.9
CA39c
51.1
At5g08690
At5g17400
55.3
At1g19330
57.0
At1g19850
At1g20620
57.5
At1g21680
58.1
At1g23440
At1g26190
58.6
At1g26630
At1g26630
At1g26850
60.3
At1g31812
pO136E2
At5g12940
61.9
At1g50420
At1g51200
65.3
At4g37120
At3g21720
68.6
pW114a
71.9
At1g77940
At3g18600
At3g18165
73.6
At3g16640
85.5
pW197d
At3g15410
87.7
At3g13000
At2g07733
92.2
At3g12560
97.2
At3g51670
At5g17400
At3g09930
At3g05165
102.2
At3g07020
At3g04740
105.5
At1g65120
At3g15310
At3g04740
110.5
114.7
At3g03960
121.1
At5g15650
128.4
At3g02200
N16
At3g58680
0.0
At1g51200
At1g63770
4.2
4.8
At5g19510
pW217c
6.0
At5g13780
At1g50030
8.1
At1g55860
At5g41900
At1g51860
9.5
At1g56145
At3g53720
11.3
At1g79950
13.1
At1g75130
15.5
17.9
At3g18165
At1g72650
19.7
pW197E2
At1g67280
At1g70610
22.7
At1g67230
At1g67170
35.8
At1g72650
47.0
At1g71010
At1g79950
63.2

## Slide 4
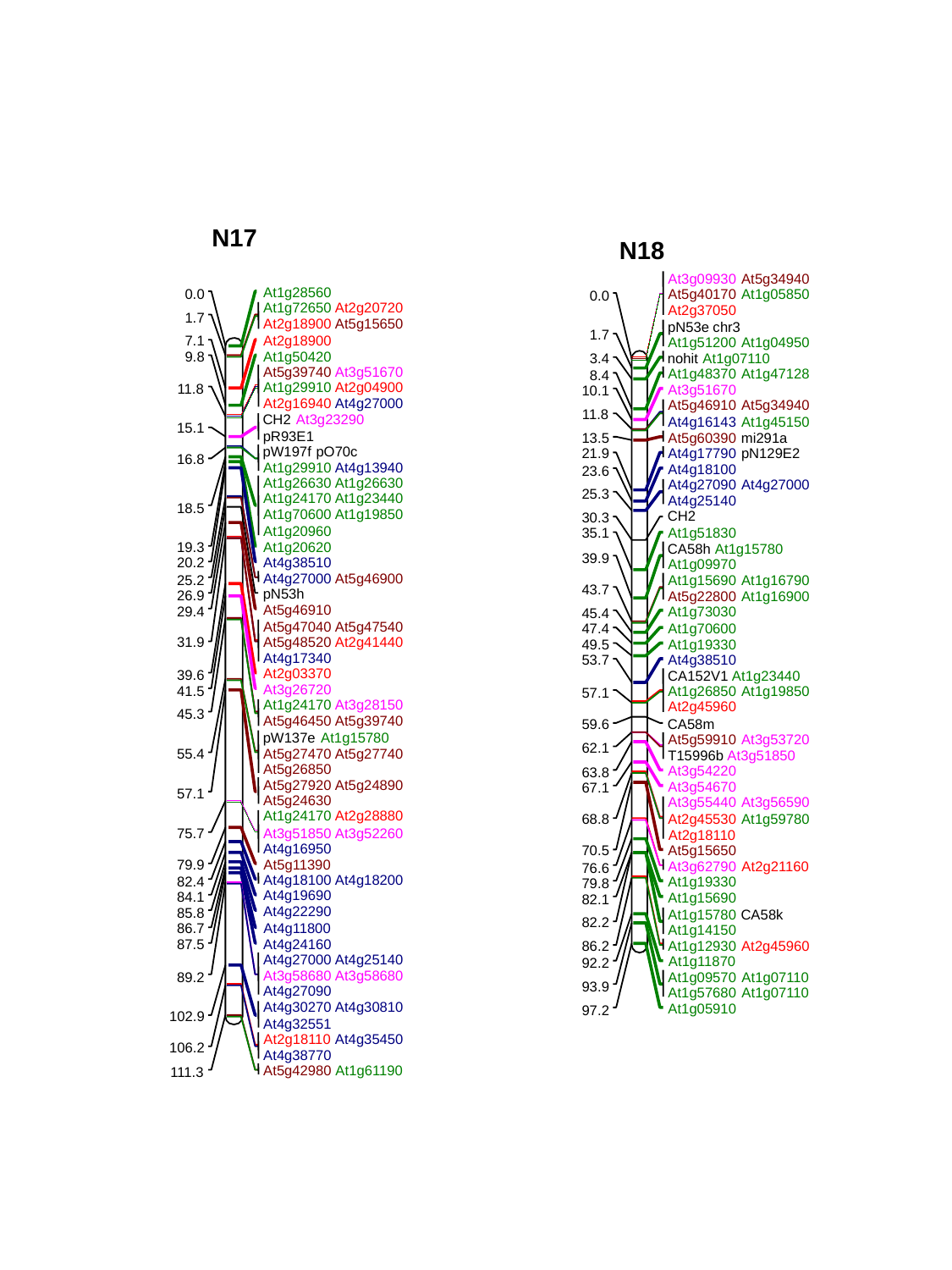

N17
At1g28560
0.0
At1g72650
At2g20720
1.7
At2g18900
At5g15650
7.1
At2g18900
9.8
At1g50420
At5g39740
At3g51670
At1g29910
At2g04900
11.8
At2g16940
At4g27000
CH2
At3g23290
15.1
pR93E1
pW197f
pO70c
16.8
At1g29910
At4g13940
At1g26630
At1g26630
At1g24170
At1g23440
18.5
At1g70600
At1g19850
At1g20960
19.3
At1g20620
20.2
At4g38510
At4g27000
At5g46900
25.2
pN53h
26.9
At5g46910
29.4
At5g47040
At5g47540
31.9
At5g48520
At2g41440
At4g17340
At2g03370
39.6
At3g26720
41.5
At1g24170
At3g28150
45.3
At5g46450
At5g39740
pW137e
At1g15780
55.4
At5g27470
At5g27740
At5g26850
At5g27920
At5g24890
57.1
At5g24630
At1g24170
At2g28880
75.7
At3g51850
At3g52260
At4g16950
79.9
At5g11390
At4g18100
At4g18200
82.4
At4g19690
84.1
At4g22290
85.8
86.7
At4g11800
87.5
At4g24160
At4g27000
At4g25140
At3g58680
At3g58680
89.2
At4g27090
At4g30270
At4g30810
102.9
At4g32551
At2g18110
At4g35450
106.2
At4g38770
At5g42980
At1g61190
111.3
N18
At3g09930
At5g34940
At5g40170
At1g05850
0.0
At2g37050
pN53e
chr3
1.7
At1g51200
At1g04950
3.4
nohit
At1g07110
At1g48370
At1g47128
8.4
At3g51670
10.1
At5g46910
At5g34940
11.8
At4g16143
At1g45150
13.5
At5g60390
mi291a
21.9
At4g17790
pN129E2
At4g18100
23.6
At4g27090
At4g27000
25.3
At4g25140
CH2
30.3
35.1
At1g51830
CA58h
At1g15780
39.9
At1g09970
At1g15690
At1g16790
43.7
At5g22800
At1g16900
At1g73030
45.4
47.4
At1g70600
49.5
At1g19330
53.7
At4g38510
CA152V1
At1g23440
At1g26850
At1g19850
57.1
At2g45960
59.6
CA58m
At5g59910
At3g53720
62.1
T15996b
At3g51850
At3g54220
63.8
At3g54670
67.1
At3g55440
At3g56590
68.8
At2g45530
At1g59780
At2g18110
70.5
At5g15650
At3g62790
At2g21160
76.6
At1g19330
79.8
At1g15690
82.1
At1g15780
CA58k
82.2
At1g14150
86.2
At1g12930
At2g45960
At1g11870
92.2
At1g09570
At1g07110
93.9
At1g57680
At1g07110
At1g05910
97.2

## Slide 5
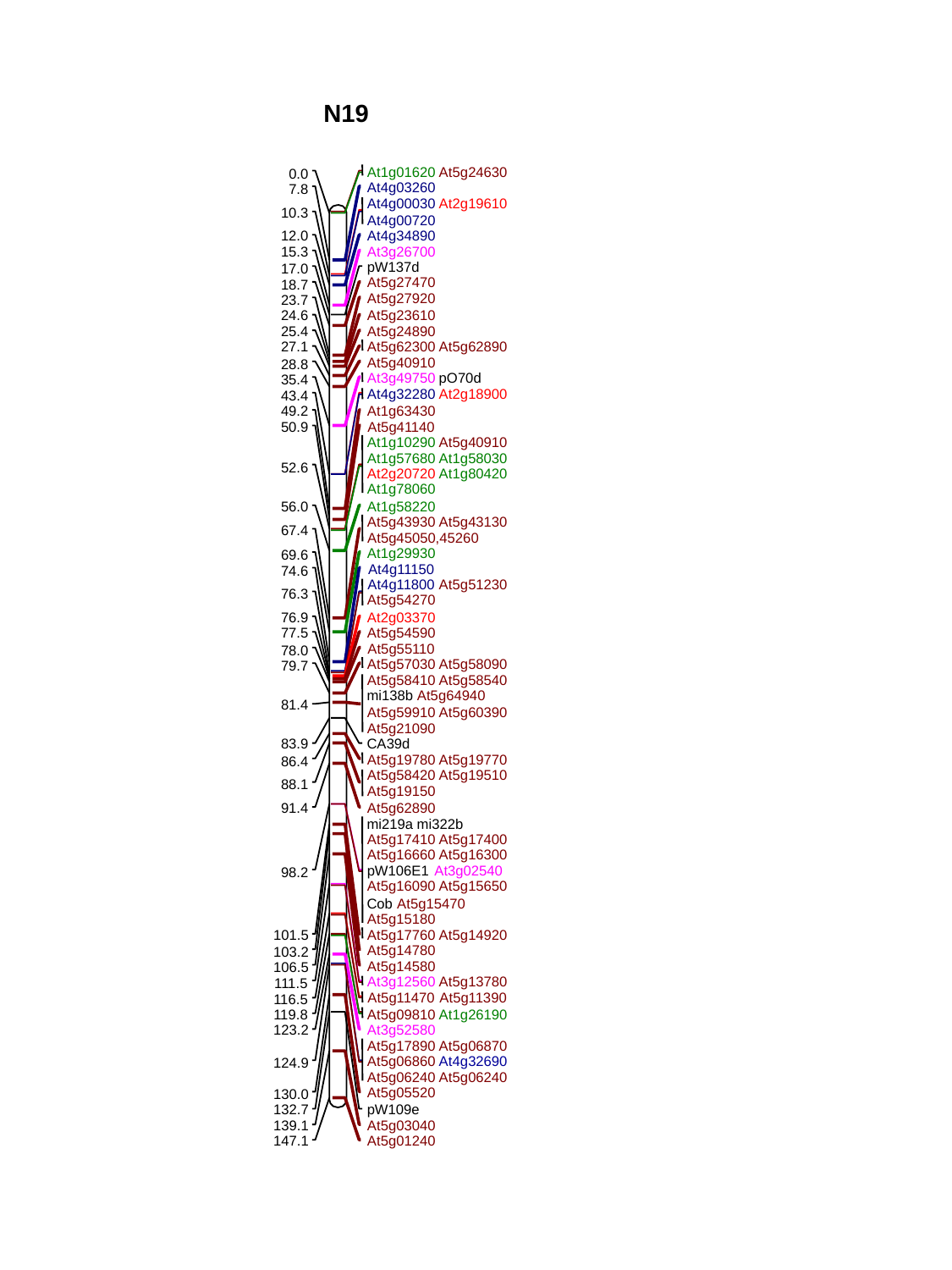

N19
At1g01620
At5g24630
0.0
At4g03260
7.8
At4g00030
At2g19610
10.3
At4g00720
12.0
At4g34890
15.3
At3g26700
pW137d
17.0
At5g27470
18.7
At5g27920
23.7
24.6
At5g23610
25.4
At5g24890
27.1
At5g62300
At5g62890
At5g40910
28.8
At3g49750
pO70d
35.4
At4g32280
At2g18900
43.4
49.2
At1g63430
50.9
At5g41140
At1g10290
At5g40910
At1g57680
At1g58030
52.6
At2g20720
At1g80420
At1g78060
56.0
At1g58220
At5g43930
At5g43130
67.4
At5g45050,45260
At1g29930
69.6
At4g11150
74.6
At4g11800
At5g51230
76.3
At5g54270
76.9
At2g03370
77.5
At5g54590
At5g55110
78.0
At5g57030
At5g58090
79.7
At5g58410
At5g58540
mi138b
At5g64940
81.4
At5g59910
At5g60390
At5g21090
83.9
CA39d
At5g19780
At5g19770
86.4
At5g58420
At5g19510
88.1
At5g19150
91.4
At5g62890
mi219a
mi322b
At5g17410
At5g17400
At5g16660
At5g16300
pW106E1
At3g02540
98.2
At5g16090
At5g15650
Cob
At5g15470
At5g15180
101.5
At5g17760
At5g14920
At5g14780
103.2
At5g14580
106.5
At3g12560
At5g13780
111.5
At5g11470
At5g11390
116.5
119.8
At5g09810
At1g26190
123.2
At3g52580
At5g17890
At5g06870
At5g06860
At4g32690
124.9
At5g06240
At5g06240
At5g05520
130.0
132.7
pW109e
139.1
At5g03040
147.1
At5g01240
