# Supplementary material for: Comparative mapping of Brassica juncea and Arabidopsis thaliana using Intron Polymorphism (IP) markers: homoeologous relationships, diversification and evolution of the A, B and C Brassica genomes
Source: BMC Genomics. 2008 Mar 3;9:113. doi: 10.1186/1471-2164-9-113 (PMC2277410; doi:10.1186/1471-2164-9-113)
Supplement: Additional file 5 — Putative ancestral Brassica karyotype (ABK2–ABK6). The file contains the figurative representation of the putative ancestral Brassica karyotype (ABK2–ABK6) predicted in our study. This is based on the conserved group structure of groups 2, 3, 4, 5 and 6 (Figure 7). Only partial organization of the putative linkage groups ABK2 and ABK3 is shown representing the common blocks shared between the three diploid Brassica genomes. [file 1471-2164-9-113-S5.ppt]

## Slide 1
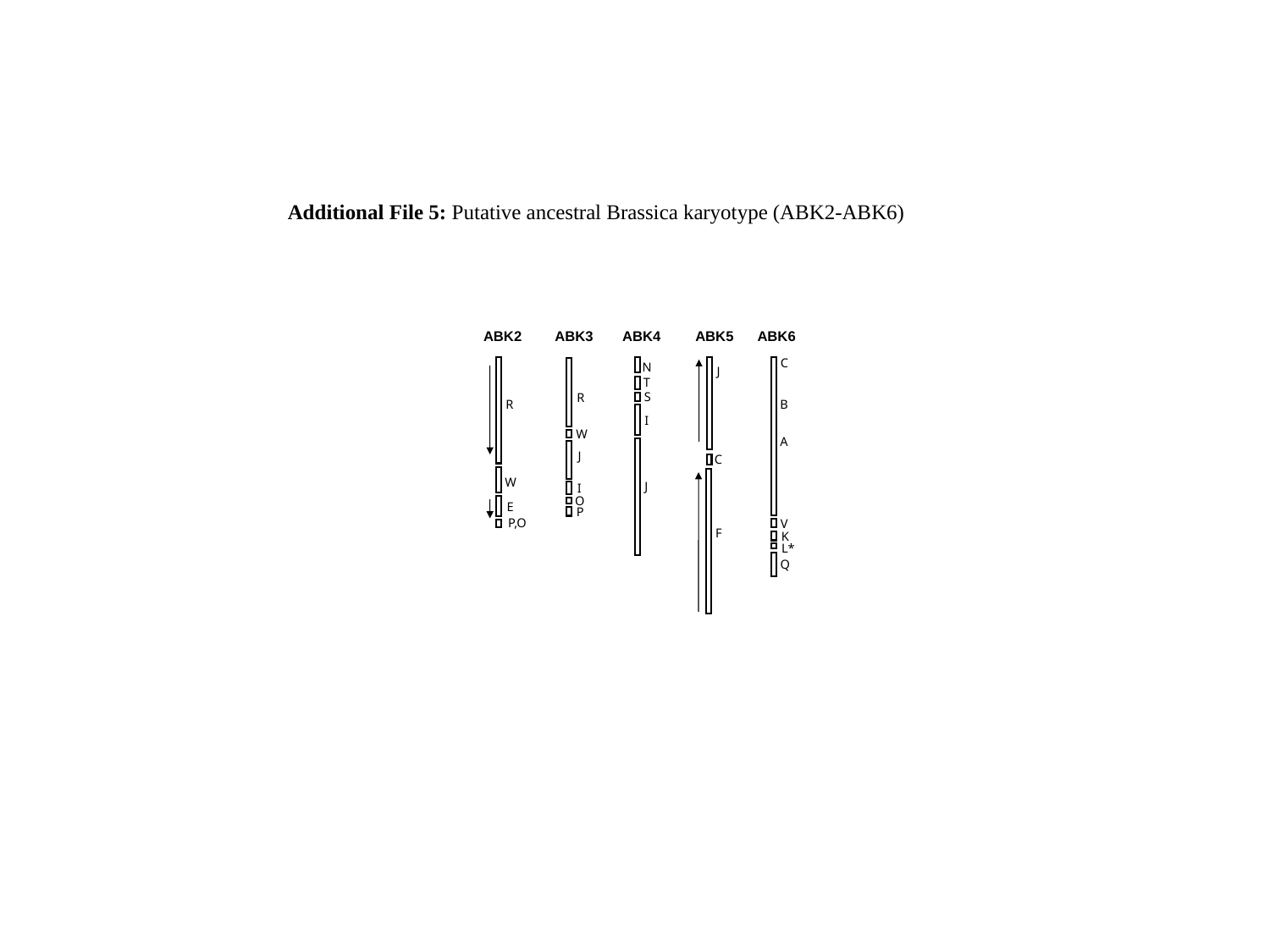

Additional File 5: Putative ancestral Brassica karyotype (ABK2-ABK6)
ABK2
ABK3
 ABK4
 ABK5
 ABK6
C
N
T
S
I
J
R
W
E
P,O
J
C
F
R
W
J
I
O
P
B
A
V
K
L*
Q
